# Exploring the role of pyroptosis in shaping the tumor microenvironment of colorectal cancer by bulk and single-cell RNA sequencing

**DOI:** 10.1186/s12935-023-02897-8

**Published:** 2023-05-18

**Authors:** Chengsheng Ding, Xiao Yang, Shuchun Li, Enkui Zhang, Xiaodong Fan, Ling Huang, Zirui He, Jing Sun, Junjun Ma, Lu Zang, Minhua Zheng

**Affiliations:** 1 Department of General Surgery, Ruijin Hospital, Shanghai Jiao Tong University School of Medicine, 197 Ruijin Er Road, Shanghai, 200025 China; 2 Shanghai Minimally Invasive Surgery Center, Ruijin Hospital, Shanghai Jiao Tong University School of Medicine, Shanghai, 200025 China; 3 Department of Surgery, Shanghai Key Laboratory of Gastric Neoplasms, Shanghai Institute of Digestive Surgery, School of Medicine, Ruijin Hospital, Shanghai Jiao Tong University, Shanghai, 200025 China

**Keywords:** Colorectal cancer, Pyroptosis, Tumor microenvironments, Immunotherapy, Single-cell analysis

## Abstract

**Background:**

Emerging studies have shown that pyroptosis plays a non-negligible role in the development and treatment of tumors. However, the mechanism of pyroptosis in colorectal cancer (CRC) remains still unclear. Therefore, this study investigated the role of pyroptosis in CRC.

**Methods:**

A pyroptosis-related risk model was developed using univariate Cox regression and LASSO Cox regression analyses. Based on this model, pyroptosis-related risk scores (PRS) of CRC samples with OS time > 0 from Gene Expression Omnibus (GEO) database and The Cancer Genome Atlas (TCGA) database were calculated. The abundance of immune cells in CRC tumor microenvironment (TME) was predicted by single-sample gene-set enrichment analysis (ssGSEA). Then, the responses to chemotherapy and immunotherapy were predicted by pRRophetic algorithm, the tumor immune dysfunction and exclusion (TIDE) and SubMap algorithms, respectively. Moreover, the Cancer Therapeutics Response Portal (CTRP) and PRISM Repurposing dataset (PRISM) were used to explore novel drug treatment strategies of CRC. Finally, we investigated pyroptosis-related genes in the level of single-cell and validated the expression levels of these genes between normal and CRC cell lines by RT-qPCR.

**Results:**

Survival analysis showed that CRC samples with low PRS had better overall survival (OS) and progression-free survival (PFS). CRC samples with low PRS had higher immune-related gene expression and immune cell infiltration than those with high PRS. Besides, CRC samples with low PRS were more likely to benefit from 5-fluorouracil based chemotherapy and anti-PD-1 immunotherapy. In novel drug prediction, some compounds such as C6-ceramide and noretynodrel, were inferred as potential drugs for CRC with different PRS. Single-cell analysis revealed pyroptosis-related genes were highly expressed in tumor cells. RT-qPCR also demonstrated different expression levels of these genes between normal and CRC cell lines.

**Conclusions:**

Taken together, this study provides a comprehensive investigation of the role of pyroptosis in CRC at the bulk RNA sequencing (RNA-seq) and single-cell RNA sequencing (scRNA-seq) levels, advances our understanding of CRC characteristics, and guides more effective treatment regimens.

**Graphical Abstract:**

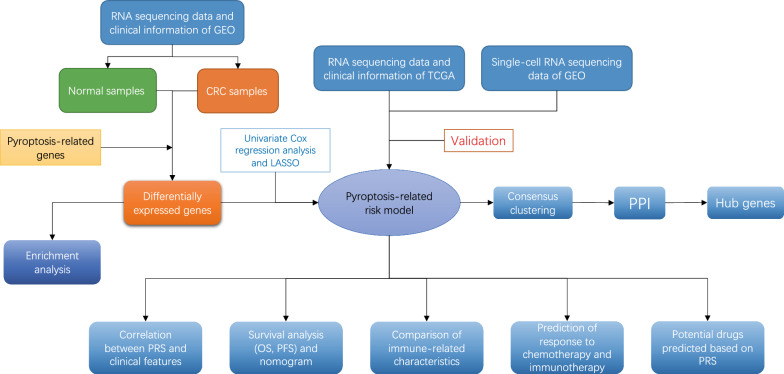

**Supplementary Information:**

The online version contains supplementary material available at 10.1186/s12935-023-02897-8.

## Background

CRC is the third most common tumor worldwide, with almost 1.9 million new cases, and its incidence closely follows breast and lung cancer. Meanwhile, it also ranks second in terms of mortality with more than 0.9 million deaths [1]. Currently, endoscopic resection, surgical resection, chemotherapy and radiotherapy are the main treatment methods for CRC. However, nearly 40% of CRC patients eventually will experience tumor relapse, while recurrence or late metastasis makes the 5-year survival rate less than 15% [2]. Hence, it is imperative to discover early diagnostic biomarkers and new therapeutic strategies for CRC.

Cell death is an indispensable way to maintain homeostasis, including apoptosis, autophagy, as well as pyroptosis [3]. Compared with other types of cell death, the most obvious disparity of pyroptosis is that it can arouse inflammation [4]. Besides, the characteristics of rapid pore formation mediated by Gasdermin family, plasma membrane rupture, cytoplasmic swelling, osmotic cell lysis, as well as DNA cleavage are observed in pyroptosis [3, 5–8]. As a type of inherently inflammatory programmed cell death (PCD), pyroptosis also induces tumor cell death through inflammasomes, which are considered as multi-protein platforms that promote caspase-1 activation and further activate the release of pro-inflammatory cytokines such as interleukin (IL)-18 and IL-1β in vitro [9]. In gastric cancer (GC), pyroptosis can be inhibited by the downregulation of gasdermin D (GSDMD), which expedites the expression of Cdk2/cyclin A2 complexes. Up-regulated Cdk2/cyclin A2 complexes can accelerate GC cell proliferation by promoting the transition from S to G2 phase [10–12].Wu et al. revealed that lipopolysaccharide-induced pyroptosis inhibits CRC tumorigenesis by promoting the expression of GSDMD and N-terminal GSDMD membrane translocation to enhance chemosensitivity of CRC cells to oxaliplatin [13]. In addition, Miguchi et al. reported that gasdermin C (GSDMC) knockdown can inhibit the proliferation of CRC cells and tumorgenesis, while increasing the expression of GSDMC can promote cell proliferation [14]. Hence, exploring the role of pyroptosis in CRC may provide a new perspective for deepening the understanding of the mechanism of CRC prognosis and anti-tumor immune activities.

In this study, pyroptosis-related risk model was developed to systematically evaluate the effect of pyroptosis on CRC. Based on PRS calculated by this model, CRC samples were divided into low- and high-risk score groups. Survival analysis revealed significant differences in prognosis between two groups, including OS and PFS. We also further used pyroptosis-related risk model to investigate the association between pyroptosis and immune-related characteristics of CRC, such as the expression of immune-related genes and infiltration of immune cells. Meanwhile, we explored pyroptosis-related risk model in the level of single cell. In terms of therapeutic prediction, we found pyroptosis-related risk model was able to distinguish CRC samples with different sensitivity to 5-fluorouracil based chemotherapy and anti-PD-1 immunotherapy. Inspired by this, some small compounds/drugs were predicted as potential salvage options in CRC samples with different PRS. This study investigated the role of pyroptosis in CRC through bulk RNA-seq and scRNA-seq, providing a novel perspective for exploring the treatment of CRC.

## Methods

### Data processing

The RNA expression profiles (Affymetrix U133Plus2) of 566 CRC samples and 19 normal colorectum tissue samples were downloaded from GSE39582 in the Gene Expression Omnibus (GEO) database (https://www.ncbi.nlm.nih.gov/geo/). According to the annotation platform, the probe matrix of each sample was processed into gene symbol data. If more than one probe corresponds to the identical gene symbol, mean value would be used as the expression of the gene. Meanwhile, the corresponding clinical features, such as OS, PFS, pathological stages and AJCC-TNM stages, were also obtained. In addition, the RNA-Seq data profiles (HTSeq-FPKM workflow type) and clinical information of CRC samples were downloaded from The Cancer Genome Atlas (TCGA) database (https://www.cancer.gov/tcga). Simultaneously, the mutation annotation format (MAF) of 399 CRC samples was downloaded from the TCGA database. These mutation data were type of Masked Somatic Mutation processed using Mutect2 software.

In Molecular Signatures Database (MSigDB) (https://www.gsea-msigdb.org/), we downloaded gene sets from GOBP_PYROPTOSIS and REACTOME_PYROPTOSIS. In addition, we collected pyroptosis-related genes from previous study [15]. After merging, a total of 57 pyroptosis-related genes were retained for subsequent analysis in this study. For reducing the batch effect, the RNA expression data from the GEO cohort and the TCGA cohort were processed by “sva” R package. In this study, CRC samples with intact information of OS (OS time and survival state) were included for further analysis. And samples whose OS time was 0 were excluded.

### Enrichment analysis based on the DEGs between CRC and normal samples

In the GEO cohort, we utilized “limma” R package to compare the expression levels of the pyroptosis-related genes between 566 CRC samples and 19 normal samples. The pyroptosis-related genes with false discovery rate (FDR) < 0.05 were considered DEGs. The expression of DEGs was displayed as a heatmap using “pheatmap” R package. After converting to Entrez Gene ID through “org.Hs.eg.db” R package, we utilized “clusterProfiler” R package to perform GO and KEGG enrichment analysis on these DEGs. Visualizing the results of GO and KEGG enrichment analysis was conducted by “enrichplot” and “ggplot2” R packages. The criterion for significant difference is the adjusted *p* value < 0.05.

### Construction of pyroptosis-related risk model

In this study, the GEO cohort served as the training group, and the TCGA cohort served as the validation group. We combined expression data of DEGs with corresponding prognostic information based on the ID of the sample. In the training group, univariate Cox regression analysis was performed to screen the DEGs that related with prognosis. The hazard ratio (HR) of each DEG was calculated, and *p* value < 0.05 was considered to be associated with prognosis. Based on prognostic DEGs, we utilized “glmnet” R package to conduct LASSO Cox regression analysis. The lambda value was determined by tenfold cross-validation. And we took lambda.min (where minimum error observed) to screen variables for developing a pyroptosis-related risk model. The following formula was used to calculate the PRS of all CRC samples: PRS = $\sum_{1}^{i}(\mathrm{Coef}i*\mathrm{Exp}i)$, where “Coef*i*” was the regression coefficient of each gene calculated by the LASSO Cox regression analysis and “Exp*i*” represented the expression values of genes from pyroptosis-related risk model.

### Survival analysis of low- and high-risk score groups

The median PRS was used as the cut-off value, all samples were divided into high- and low-risk score groups. Principal component analysis (PCA) was performed by “limma” R package to verify whether the risk model could distinguish high- and low-risk score groups. We compared prognosis of CRC samples between high- and low-risk score groups using the Kaplan–Meier method via the “survival” R package. For evaluating the accuracy of pyroptosis-related risk model in OS prediction, the time-dependent receiver operating characteristic curves (ROC) were plotted via “timeROC” R package. The area under the ROC curve (AUC) indicates the accuracy of the prediction. Compared with AUC = 0.5, the AUC p values of 1,3,5-year survival prediction were calculated by “pROC” and “verification” R packages. In the validation group, the same cut-off value as the training group was used to divide all CRC samples to high- and low-risk score groups. The same method was applied to validate the reliability and applicability of pyroptosis-related risk model in prognosis prediction. In addition, we utilized “maftools” R package to explore the gene mutation of pyroptosis-related risk model and the difference of gene mutation between high- and low-risk score groups.

### The distribution of PRS in different clinical features

In the GEO and TCGA cohorts, we combined the PRS with the clinical information of the corresponding CRC samples, including age, gender, AJCC-TNM stages, and pathological stages. Clinical information of CRC samples was demonstrated in Additional file 2: Table S1. Beyond that, we obtained the status data of the CpG island methylator phenotype (CIMP), chromosomal instability (CIN), DNA mismatch repair (MMR), and *KRAS*/*BRAF*/*TP53* mutation in CRC samples from the GEO cohort. Meanwhile, based on RNA-seq data, “CMScaller” R package was applied to predict the Consensus Molecular Subtype (CMS) of CRC samples from the GEO cohort. Finally, the comparison of PRS between subgroups was conducted by “limma” R package.

### Construction of a nomogram

Univariate and multivariate Cox regression analyzes were first performed to identify independent indicators associated with OS. Based on these independent indicators, we utilized “regplot” and “survival” R packages to develop nomogram for 1-, 3-, and 5-year OS predictions. Then, the accuracy of the nomogram in OS prediction was verified by time-dependent calibration curves using “rms” R package. Finally, “survivalROC” R package was used to plot the ROC curves of single independent prognostic indicator and nomogram, and AUC was calculated to compare the value in predicting OS of CRC.

### Gene set enrichment analysis (GSEA)

GSEA, a knowledge-based approach for interpreting genome-wide expression profiles, can be used to explore biological functions and pathways [16]. In this study, we conducted GSEA using “clusterProfiler” R package to compare the differences in biological characteristics between CRC samples from high- and low-risk score groups, the pathways with p < 0.05 were consider statistically different. The “h.all.v7.4.symbols.gmt” was downloaded from Molecular Signatures Database (MSigDB) as the reference gene set.

### Comparison of features between high- and low-risk score groups

According to the studies of Barbie et al. [17, 18], this study collected 3 gene sets corresponding to antigen presentation, immune-activation and immune-checkpoint, respectively. The expression level of these genes was compared between low- and high-risk score group. ESTIMATE (Estimation of STromal and Immune cells in MAlignant Tumor tissues using Expression data) is an algorithm that can predict tumor purity, and the infiltration of stromal/immune cells in tumor tissues by expression data. Based on RNA-seq data, we evaluated the stromal score, immune score and tumor purity of each CRC sample via “estimate” R package. The ssGSEA was performed by the "GSVA" R package to quantify the relative abundance of immune cell infiltration in the CRC TME. The reference gene sets were downloaded from TISIDB: an integrated repository portal for tumor–immune system interactions, which included 28 types of human immune cells such as activated CD8+ T cell, activated CD4+ T cell, gamma delta T cell, as well as regulatory T cell [19].

### Prediction of therapeutic response in CRC samples with different PRS

In the aspect of chemotherapy, the half maximal inhibitory concentration (IC50) of 5-fluorouracil was calculated by pRRophetic algorithm. And the value of IC50 between high- and low-risk score groups was compared. The potential response of CRC samples to immunotherapy were inferred by TIDE (http://tide.dfci.harvard.edu/) and SubMap (https://cloud.genepattern.org/gp) algorithms. Based on the annotation file subtypes data [20], the response of two groups’ samples to anti-PD-1/ CTLA4 immunotherapy was predicted and compared. To further validate the feasibility of PRS in the prediction of response to immunotherapy, we also utilized “IMvigor210CoreBiologies” R package to obtain RNA-seq data and clinical information of IMvigor 210 cohort, which is a clinical trial about Atezolizumab in patients with bladder urothelial carcinoma. The PRS of each sample in this cohort was calculated based on pyroptosis-related risk model, and then the PRS among samples with different immune characteristics was compared.

### Prediction of potential drug for CRC

In order to explore novel drug treatment strategies for CRC, we further screened potential compounds/drugs that may be effective against cancer based on the PRS of CRC samples. According to study of Yang [21], we downloaded expression profile data and somatic mutation data of human cancer cell lines (CCLs) from the Broad Institute Cancer Cell Line Encyclopedia (CCLE) project [22]. Both the Cancer Therapeutics Response Portal (CTRP; https://portals.broadinstitute.org/ctrp) and PRISM Repurposing dataset (PRISM, https://depmap.org/portal/prism/) were used to obtain the drug sensitivity data of CCLs. There are the sensitivity data for 481 compounds over 835 CCLs and 1448 compounds over 482 CCLs in CTRP and PRISM, respectively. And sensitivity of drug was quantified by the area under the dose–response curve (AUC), increased sensitivity to drug was represented by lower AUC. K-nearest neighbor (k-NN) imputation was utilized to identify and replace missing values of AUC prior to drug sensitivity prediction. Before imputation, we excluded compounds with more than 20% missing data.

### Single-cell RNA sequencing (scRNA-seq) analysis

In this study, we downloaded scRNA-seq data of GSE132257 from GEO database. First, scRNA-seq data was filtered and standardized using “Seurat” R package. The genes with large variance were reserved for subsequent analysis. PCA was then conducted to reduce dimensionality of these genes. And t-SNE was applied to sort cells into different clusters. The cell annotation of each cluster was conducted by “SingleR” and “celldex” R packages with reference to HumanPrimaryCellAtlasData. In order to calculate the activity of pyroptosis-related genes in cells, we utilized to “AUCell” R package to calculate the AUC of each cell with the reference to genes in pyroptosis-related risk model and then mapped the AUC to the corresponding cell. Cells that express more genes from pyroptosis-related risk model will exhibit higher AUC values than cells expressing fewer genes.

### Cell lines culture and Quantitative real-time polymerase chain reaction (RT-qPCR)

All cell lines NCM-460, HT-29, HCT116 were obtained from the American Type Culture Collection (ATCC, Manassas, VA) and stored at the Shanghai Institute of Digestive Surgery. Cells were cultured in RPMI-1640 or McCoy's 5A (Gibco, Grand Island, NY, USA) supplemented with 10% fetal bovine serum (FBS), 1% penicillin/streptomycin (Gibco) at 37 °C with 5% CO2. The total RNAs from CRC cell lines were extracted with the Trizol reagent (Invitrogen, CA, USA). Then, we utilized the NanoDrop 2000 spectrophotometer (Thermo) to quantify RNA, and reverse transcribed RNA to cDNA by HiScript® RT SuperMix for qPCR with gDNA wiper (Vazyme, China). Finally, RT-qPCR was performed using ChamQ Universal SYBR qPCR Master Mix (Vazyme, China) according to the cycler protocol (5 min at 95 °C, 40 cycles of 15 s at 95 °C, 60 s at 60 °C, and 5 min at 72 °C). GAPDH was exploited as an internal reference. The mRNA relative expression of genes was detected by 2^−ΔΔCt^ methods. The primer sequences used for analysis are listed in Table 1.

**Table 1:** Primer sequences of genes in pyroptosis-related risk model

Gene (Gene ID)	Primer sequence
CHMP6 (79643)	F: AAGGCCATCCTGCAACTGAAG
	R: GCTGCTCCTGGTATCGCTT
GSDMD (79792)	F: GTGTGTCAACCTGTCTATCAAGG
	R: CATGGCATCGTAGAAGTGGAAG
GZMB (3002)	F: CCCTGGGAAAACACTCACACA
	R: GCACAACTCAATGGTACTGTCG
NLRP1 (22861)	F: GCAGTGCTAATGCCCTGGAT
	R: GAGCTTGGTAGAGGAGTGAGG
CYCS (54205)	F: CTTTGGGCGGAAGACAGGTC
	R: TTATTGGCGGCTGTGTAAGAG
CASP3 (836)	F: CATGGAAGCGAATCAATGGACT
	R: CTGTACCAGACCGAGATGTCA
CASP1 (834)	F: TTTCCGCAAGGTTCGATTTTCA
	R: GGCATCTGCGCTCTACCATC
CASP6 (839)	F: ATGGCGAAGGCAATCACATTT
	R: GTGCTGGTTTCCCCGACAT
GAPDH (2597)	F: GGAGCGAGATCCCTCCAAAAT
	R: GGCTGTTGTCATACTTCTCATGG

### Consensus clustering and Protein–protein interaction (PPI) network

In this study, “limma” R package was utilized to identify the DEGs between high- and low-risk score groups, and FDR < 0.01 was considered statistically significant. Based on these DEGs, CRC samples were clustered into different clusters by “ConsensusClusterPlus” R package. We utilized “Rtsne” R package to perform t-distributed stochastic neighbor embedding (t-SNE) to verify distribution differences between clusters, and survival analysis between clusters were performed. Gene set variation analysis (GSVA) was performed to predict the biological features and immune-related characteristics between different clusters by “GSVA” R package. Furthermore, based on the characteristics of different clusters, we explored their relationship with CMS subgroups.

Immune genes were downloaded from the ImmPort (www.immport.org) based on overlapping genes in immune genes and DEGs, PPI network was constructed with high confidence (0.700) via STRING online database (https://string-db.org/). The PPI network was further processed using Cytoscape software. The top 10 hub genes were screened by cytoHubba, a plug-in of Cytoscape. The association between 10 hub genes and immune cells was explored. We utilized the "survival" R package to verify whether the expression levels of 10 hub genes are associated with the prognosis of CRC samples. Finally, TIMER2.0 (Tumor IMmune Estimation Resource; http://timer.cistrome.org/) was utilized to explore the relation between prognostic hub genes and immune cells infiltration.

### Statistical analysis

Wilcoxon rank-sum test was utilized to compare differences between two groups, whereas the Kruskal–Wallis test was made to compare three or more groups. Kaplan–Meier method was applied to identify the survival differences between high- and low-risk score groups. Univariate and multivariate Cox regression analyses were performed to determine independent prognostic indicators in CRC. The ROC curves were plotted to access the prognostic value of each indicator and nomogram. All the statistical analyses were conducted in R software (version 4.0.3).

## Results

### Exploration of differences between CRC and normal samples

**Fig. 1:**
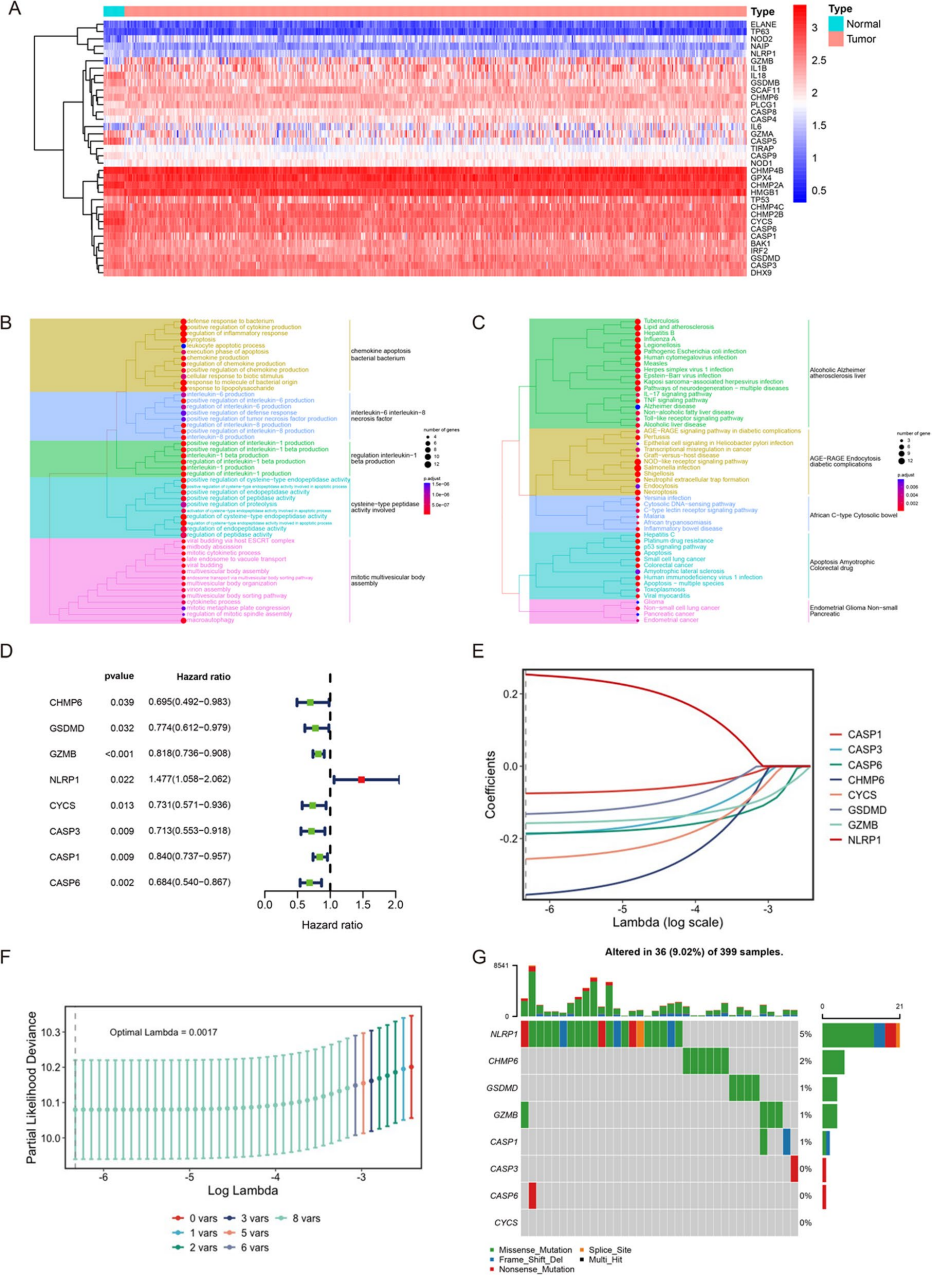
Construction of pyroptosis-related risk model. **A** 35 DEGs between CRC and normal samples in the GEO cohort. **B** The results of GO enrichment analysis on DEGs. **C** The results of KEGG enrichment analysis on DEGs. **D** 8 prognostic DEGs selected by univariate Cox regression analysis. **E** Construction of pyroptosis-related risk model by LASSO Cox regression analysis. **F** The number of genes determined when lambda value is lambda.min (0.00178). The dashed line indicates selected lambda value. **G** The landscape of mutation of genes after LASSO Cox regression analysis

In this study, 52 pyroptosis-related genes were retained for subsequent analysis after reducing the batch effect between the GEO and TCGA cohorts. Additional file 1: Fig. S1A showed a clear boundary between two cohorts before batch effect reduction. However, Additional file 1: Fig. S1B showed no significant batch effect between the two cohorts after treatment. By comparing the expression of these genes between CRC and normal samples, we identified 35 genes with FDR < 0.05 in the GEO cohort. These genes were called DEGs and displayed in the form of heat map. As shown in Fig. 1A, there were 13 up-regulated genes and 22 down-regulated genes in CRC samples. The GO enrichment analysis revealed DEGs played a role in some important biological processes, such as regulation of inflammatory response, pyroptosis, cytokine production, response to molecules of bacterial origin, response to lipopolysaccharide and interleukin production. Beyond that, these DEGs also had a complex set of molecular function such as regulation of endopeptidase activity, and cytokine process (Fig. 1B). The results of KEGG enrichment analysis showed us that DEGs were involved in many cell and biological functions, such as multiple types of infection, NOD-like receptor signaling pathway, colorectal cancer and inflammatory bowel disease (Fig. 1C). According to the above results of enrichment analysis, we speculated that DEGs were associated with the prognosis and immunity of CRC, indicating that the expression imbalance of pyroptosis regulatoring genes may play a crucial role in the occurrence and progression of CRC via above pathways and mechanisms.

### Construction of pyroptosis-related risk model

According to the inclusion and exclusion criteria, 556 and 515 CRC samples were included from the GEO and TCGA cohorts, respectively, for subsequent analysis. Based on the above DEGs, univariate Cox regression analysis was conducted to screen out 8 DEGs related with prognosis of CRC samples. NLRP1 acted as a risk factor in CRC samples’ survival (HR = 1.477(1.058–2.062); *p* = 0.022). However, the HR of CHMP6, GSDMD, GZMB, CYCS, CASP3, CASP1, CASP6 was < 1 (*p* < 0.05), which represented protective factor for prognosis in CRC (Fig. 1D). To comprehensively evaluate each CRC sample, a pyroptosis-related risk model was developed from these 8 prognostic DEGs by LASSO Cox regression analysis (Fig. 1E, F). According to the corresponding coefficient of each gene calculated by this model, the final model was: PRS = 0.2533* NLRP1- 0.3551* CHMP6- 0.1320* GSDMD- 0.1574* GZMB- 0.2563* CYCS- 0.1878* CASP3- 0.0752* CASP1- 0.1858* CASP6. As shown in Fig. 1G, 36 out of 399 CRC samples (9.02%) experienced mutations of pyroptosis-related genes. The gene with highest mutation frequency was NLRP1, while CYCS was not mutated in CRC samples. In terms of mutation types, missense mutation was the most common mutation. Finally, PCA analysis revealed that there was a clear division between high- and low-risk score groups (Additional file 1: Fig. S1C, D). These results illustrated that pyroptosis-related risk model had the ability to distinguish CRC samples with different PRS. And this conclusion was further confirmed in the TCGA cohort (Additional file 1: Fig. S1E, F).

### Survival analysis between CRC samples with different PRS

**Fig. 2:**
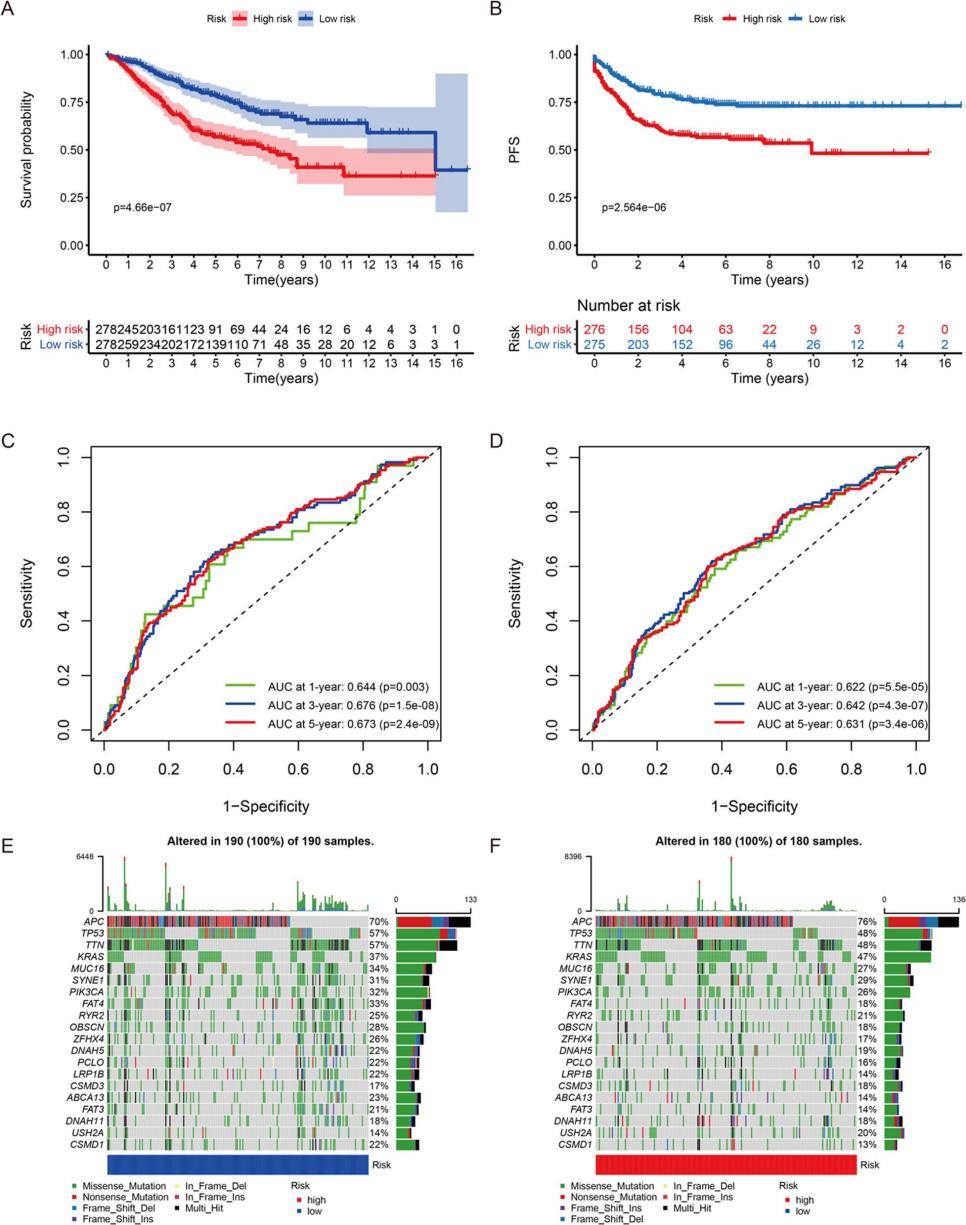
The differences of prognosis between high- and low-risk score groups in the training group. **A** The comparison of OS between high- and low-risk score groups. **B** The comparison of PFS between high- and low-risk score groups. **C** The ROC curves for OS prediction by PRS at 1-, 3- and 5-year. **D** The ROC curves for PFS prediction by PRS at 1-, 3- and 5-year. **E** The landscape of genetic mutation in the low-risk score group. **F** The landscape of genetic mutation in the high-risk score group

In the training group, all CRC samples were divided into high- and low-risk score groups based on the cut-off value (− 7.052). Survival analysis demonstrated that CRC samples in high-risk score group had poorer OS and PFS (Fig. 2A, B). Subsequently, time-dependent ROC curves were plotted to test the accuracy of PRS in predicting OS and PFS. As shown in the Fig. 2C, AUC at 1-, 3- and 5-year was 0.644 (p = 0.003), 0.676 (p = 1.5e-08) and 0.673 (p = 2.4e-09), respectively. It meant that PRS had value in predicting OS. Similarly, Fig. 2D illustrated that PRS could act as a reliable indicator to predict PFS. As expected, PRS still had the ability to predict CRC samples’ OS and PFS accurately in the validation group (Additional file 1: Fig. S2A–D). And genetic mutation overviews of 190 low-risk CRC samples and 180 high-risk CRC samples were displayed in Fig. 2E and F, respectively. *APC*, *TP53*, *TTN*, *KRAS*, as well as *MUC16* were top 5 genes with mutation frequency. Nonsense mutation was the most common mutation type in *APC*, and the most common mutation type in other genes was missense mutation.

### The association between PRS and clinical features

**Fig. 3:**
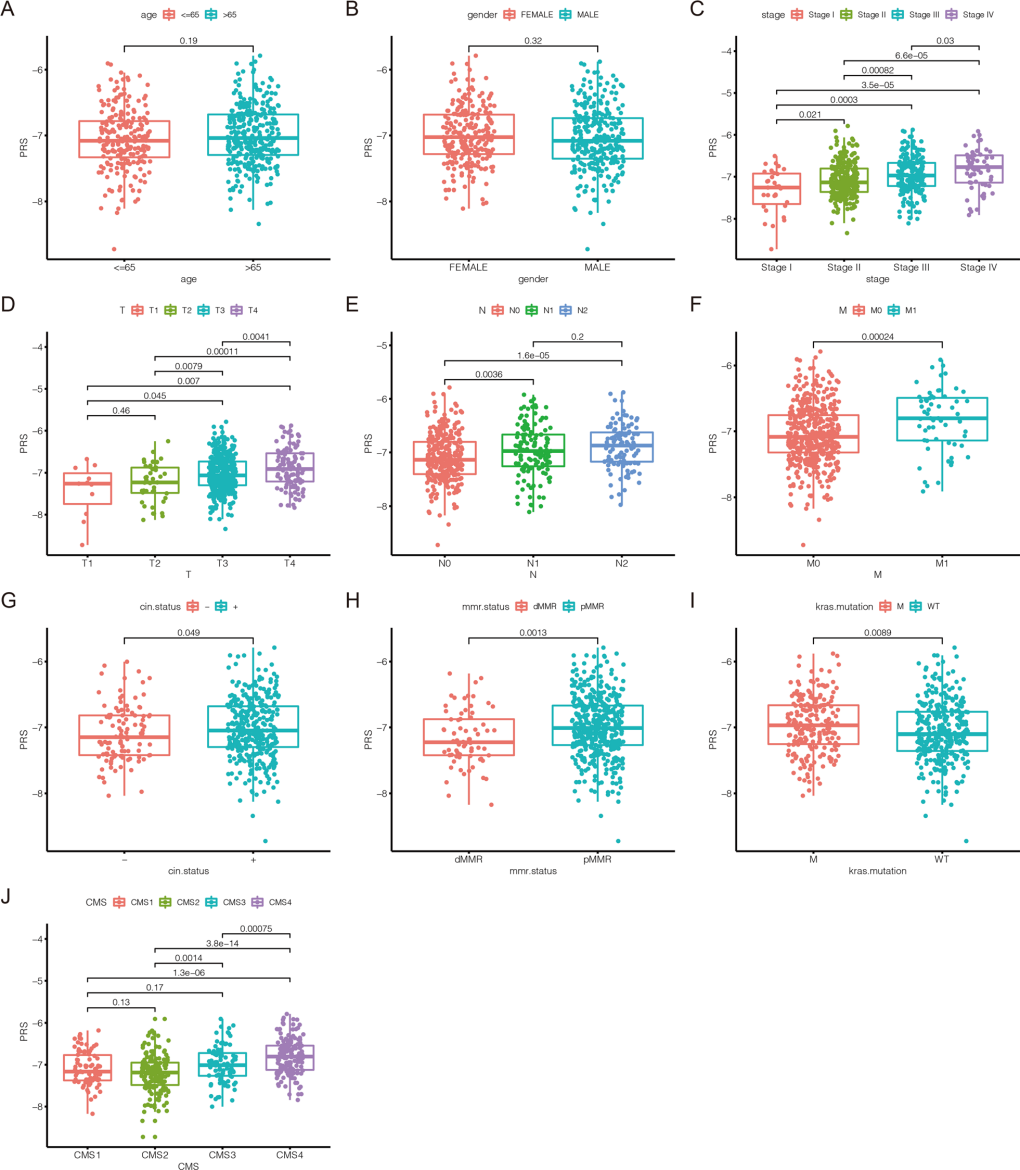
The association between PRS and clinical features in the GEO cohort. **A** The comparison of PRS in CRC samples’ age. **B** The comparison of PRS in CRC samples’ gender. **C** The comparison of PRS in CRC samples’ pathological stages. **D** The comparison of PRS in CRC samples’ AJCC-T stages. **E** The comparison of PRS in CRC samples’ AJCC-N stages. **F** The comparison of PRS in CRC samples’ AJCC-M stages. **G** The comparison of PRS between CIN (−) and CIN (+) subgroups. **H** The comparison of PRS between CRC samples with dMMR and pMMR. **I** The comparison of PRS between *KRAS* wild-type and *KRAS* mutant CRC samples. **J** The comparison of PRS between CMS subgroups

**Fig. 4:**
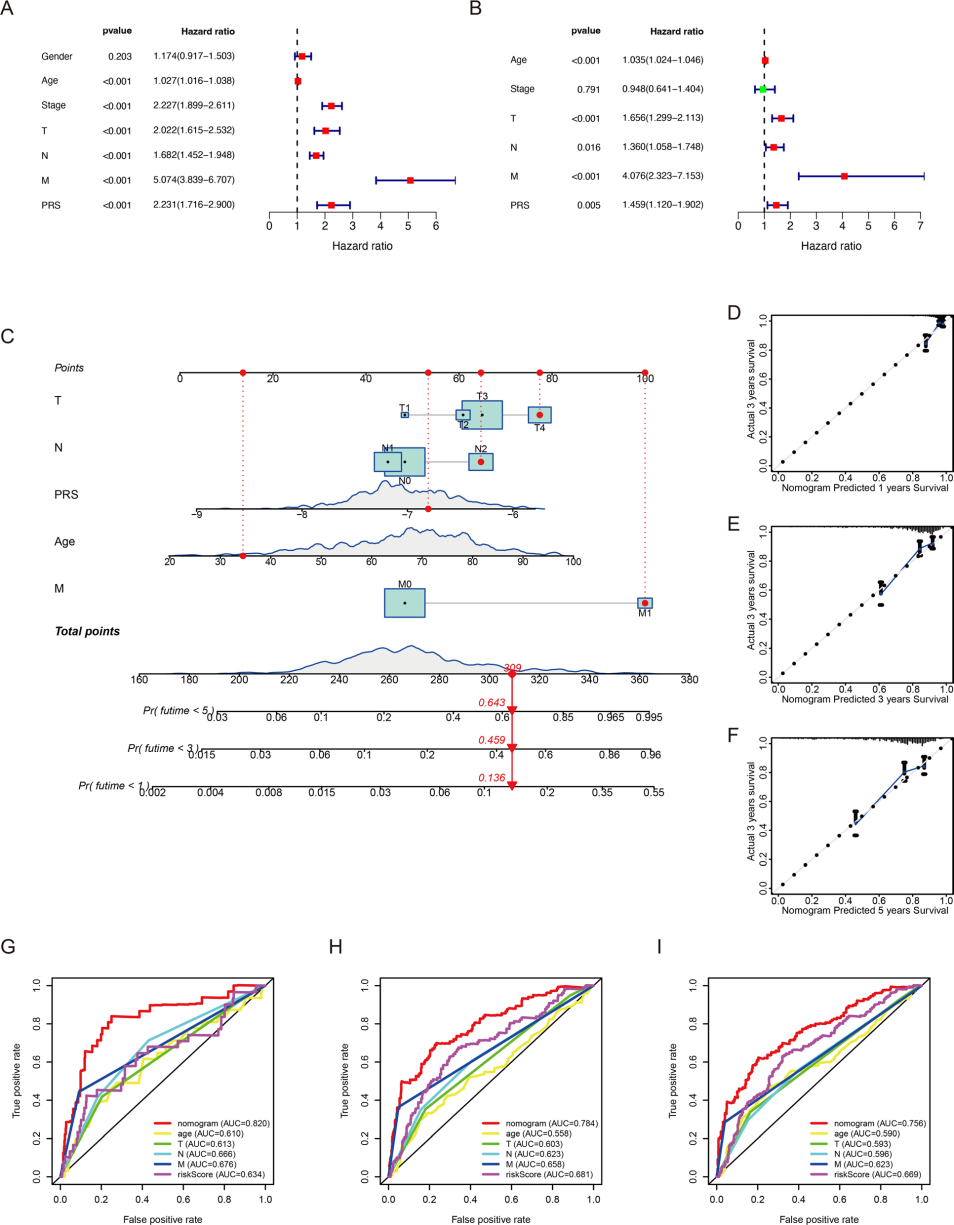
A nomogram for survival prediction in CRC. **A** Univariate Cox regression analysis to identify risk factors in CRC survival. **B** Multivariate Cox regression analysis to identify independent prognostic indicators in CRC. **C** A nomogram composed of independent prognostic indicators. **D**–**F** The calibration curve of nomogram at 1-year, 3-year and 5-year, respectively. **G**–**I** Time-dependent ROC curves of nomogram and independent prognostic indicators in OS prediction at 1-year, 3-year and 5-year, respectively

In this study, the distribution of PRS in different clinical subgroups was investigated. In the GEO cohort, we found that there was no significant difference in the distribution of PRS in age and gender (Fig. 3A, B). However, CRC samples’ PRS was associated with tumor progression. The PRS of samples with advanced tumor was significantly higher than that of samples with early tumor (Fig. 3C). We also found that CRC samples with higher AJCC-TNM stages had higher PRS (Fig. 3D–F). The same results were confirmed in the TCGA cohort (Additional file 1: Fig. S3). This means that CRC samples with high PRS tend to be in advanced stage, which also partly explains why CRC samples in the high-risk score group have a poor prognosis. PRS also increased significantly in CIN (+) subgroup (*p* = 0.049; Fig. 3G). Compared with proficient MMR (pMMR) subgroup, CRC samples with deficient MMR (dMMR) had lower PRS, which indicated CRC with low PRS may be sensitive to immunotherapy (*p* = 0.0013; Fig. 3H). CRC samples with *KRAS* mutation had higher PRS (*p* = 0.0089; Fig. 3I). However, we did not observe significant difference in PRS between the CIMP, *BRAF*/*TP53* mutation subgroups (Additional file 1: Fig. S4A–C). Beyond that, we predicted the CMS of CRC samples based on the RNA expression data in the GEO cohort (Additional file 1: Fig. S5A). As demonstrated in the Additional file 1: Fig. S5B, 82 CRC samples were classified into CMS1 which was characterized by microsatellite instability (MSI). 160 samples were divided into CMS2 which was characterized by microsatellite stability (MSS). 89 samples were divided into CMS3 which was characterized by differentiation and fatty acids metabolism. And 160 samples were divided into CMS4 which was characterized by epithelial-mesenchymal transition (EMT). The distribution of PRS in 4 CMS subgroups was displayed in the Fig. 3J. In the GEO cohort, the result of univariate Cox regression analysis showed age, pathological stages, AJCC-TNM stages and PRS were risk factors for CRC (Fig. 4A). Subsequent multivariate Cox regression analysis illustrated that only age, AJCC-TNM stages and PRS could be used as independent prognostic indicators (Fig. 4B). Furthermore, as shown in Additional file 1: Fig. S6, PRS was also confirmed to be an independent prognostic indicator TCGA cohorts. Based on these independent prognostic indicators, we developed a nomogram to evaluate 1-, 3-, and 5-year survival rates in CRC samples (Fig. 4C). Time-dependent calibration curves revealed that nomogram had a high accuracy in predicting prognosis of CRC (Fig. 4D–F). In addition to this, the AUC at 1-,3-,5-year demonstrated nomogram had a better predictive ability than a single prognostic indicator (Fig. 4G–I).

### The differences of biological features between high- and low-risk score groups

**Fig. 5:**
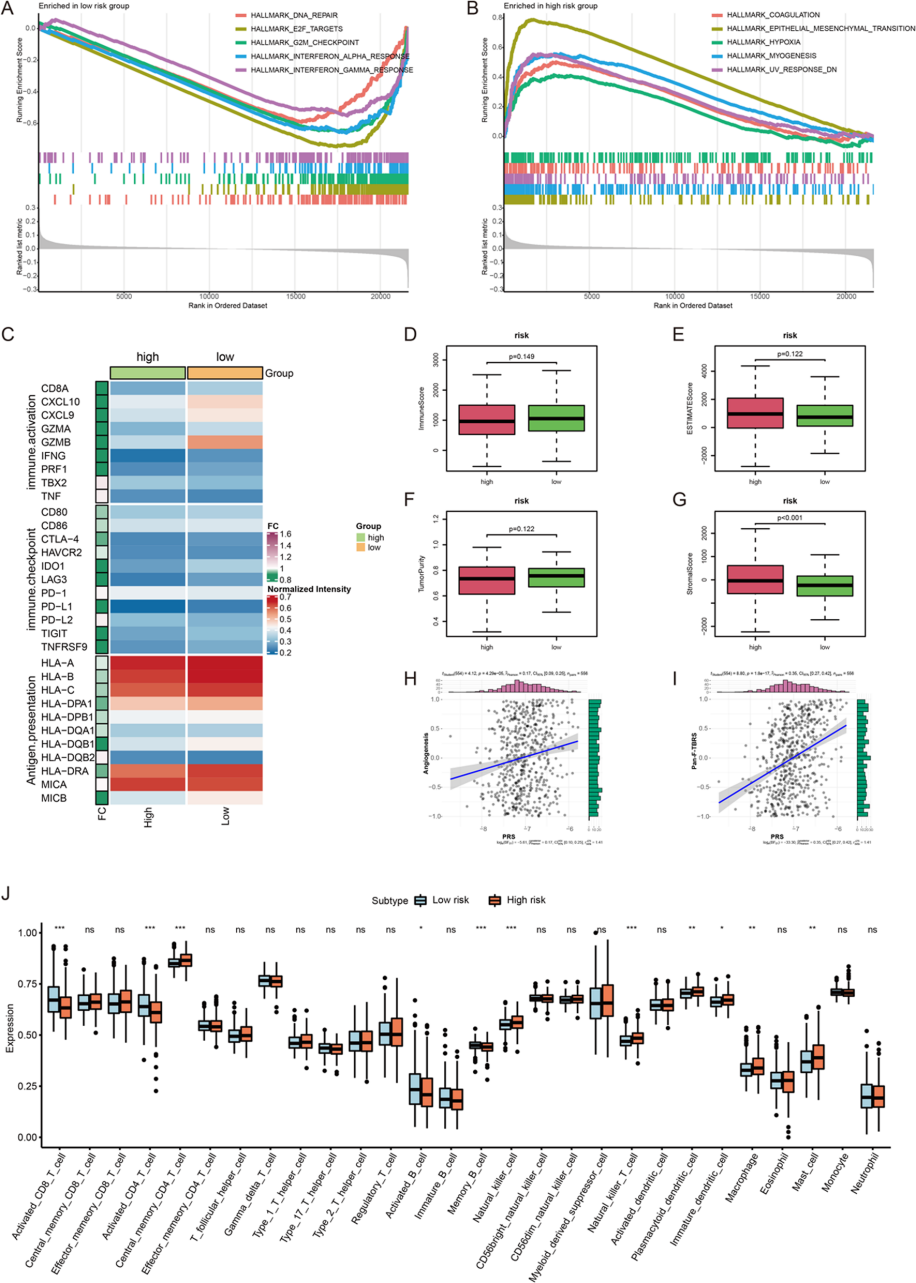
The differences of biological features between high- and low-risk score groups. **A** The hallmarks of cancer enriched in low-risk score group. **B** The hallmarks of cancer enriched in high-risk score group. **C** The comparison of expression level of antigen presentation, immune-activation and immune-checkpoint gene sets between high- and low-risk score groups. **D** The comparison of immune score between high- and low-risk score groups. **E** The comparison of estimate score between high- and low-risk score groups. **F** The comparison of tumor purity between high- and low-risk score groups. **G** The comparison of stromal score between high- and low-risk score groups. **H** The correlation between PRS and activity of angiogenesis. **I** The correlation between PRS and activity of Pan-F-TBRS. **J** Abundance of 28 immune cells in TME. ***p < 0.001; **p < 0.01; *p < 0.05; ns: no significance

After GSEA, we found that DNA repair, E2F targets, G2M checkpoint, interferon alpha response, as well as interferon gamma response were enriched in low-risk score group (Fig. 5A). Coagulation, epithelial mesenchymal transition, hypoxia, myogenesis and down-regulated response to ultraviolet (UV) were enriched in high-risk score group (Fig. 5B). These results indicated that there may be differences in immune characteristics between the high- and low-risk score groups. As shown in Fig. 5C, we found that almost all immune-related genes were highly expressed in CRC samples from low-risk score group. Although there was no significant difference of immune score, estimate score and tumor purity between high- and low-risk score groups, we found CRC samples with high PRS had higher stromal score (Fig. 5D–G). Stromal score represented the abundance of stromal cells in the tumor sample. Therefore, high stromal score was conducive to the growth and metastasis of tumor cells, and also affected the anti-tumor immune effect. Meanwhile, we also explored the correlations of PRS with stroma-related activities, including angiogenesis and pan-fibroblast TGF-β response signature (Pan-F-TBRS). As demonstrated in Fig. 5H and I, there were positive correlations between PRS and angiogenesis (*r* = 0.17, *p* = 4.29e−05) and Pan-F-TBRS (*r* = 0.35, *p* = 1.8e−17). In the assessment of immune cell infiltration, we found there were significant differences of immune cell infiltration between high- and low-risk score group (Fig. 5J). Activated CD8+ T cell, activated CD4+ T cell, activated B cell and memory B cell infiltrated more in the CRC samples from low-risk score group. Meanwhile, the infiltrations of natural killer cell, natural killer T cell, plasmacytoid dendritic cell, immature dendritic cell, macrophage and mast cell were higher in the high-risk score group. These results indicated that CRC samples with different PRS perhaps had different sensitivity to immunotherapy.

### Evaluation and prediction of therapy strategies in CRC

**Fig. 6:**
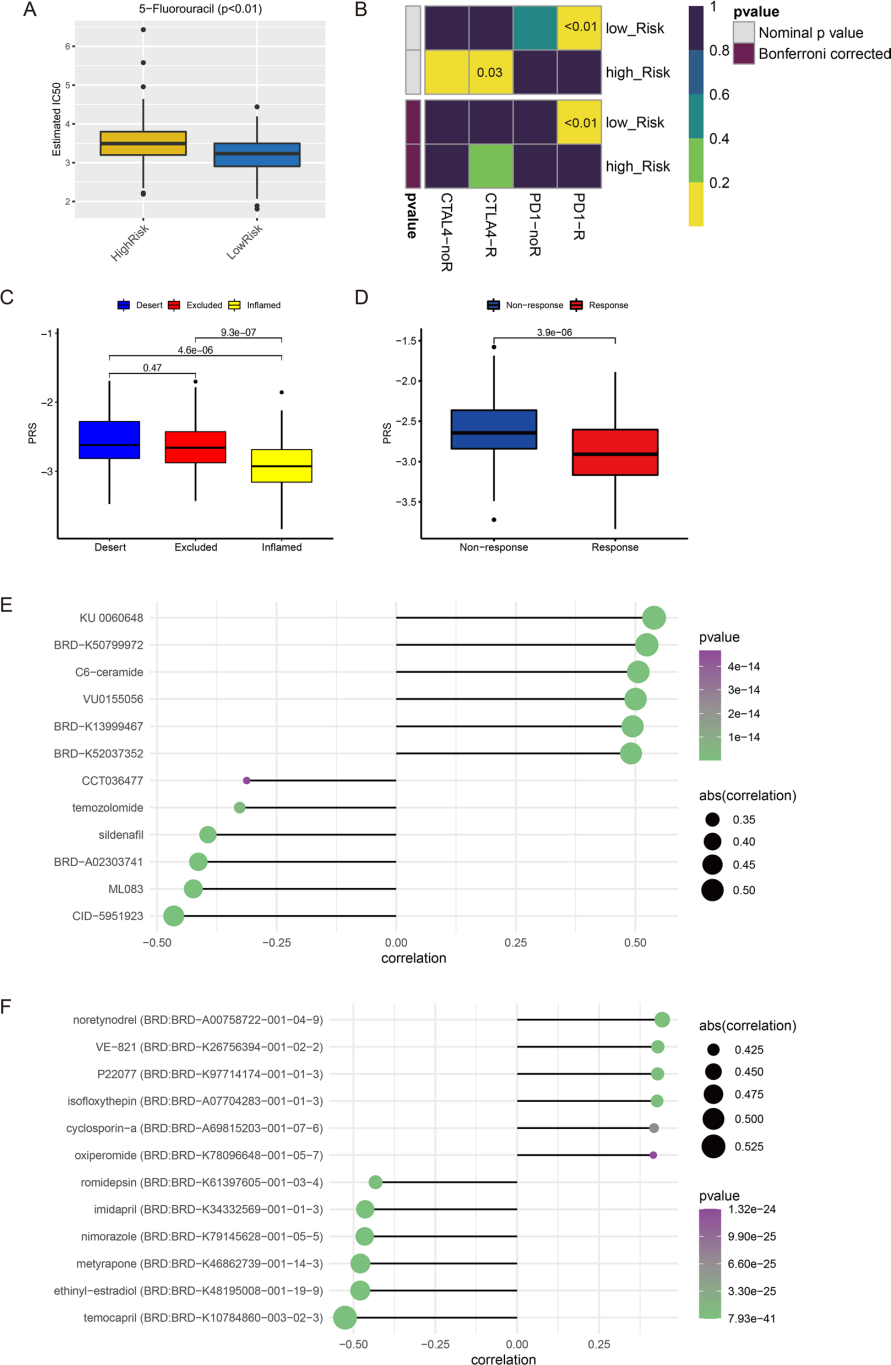
Prediction of therapy strategies in CRC. **A** Estimated IC50 of 5-fluorouracil, lower IC50 means more sensitive to 5-fluorouracil. **B** Prediction of responses of CRC samples between high- and low-risk score groups to immunotherapies. **C** The comparison of PRS between samples with different immune phenotype in IMvigor 210 cohort. **D** The comparison of PRS between samples with non-response and response to immunotherapy in IMvigor 210 cohort. **E** Predicted compounds/drugs related with PRS in CTRP. **F** Predicted compounds/drugs related with PRS in PRISM

According to the comparison of IC50 value between high- and low-risk score groups, CRC samples with high PRS had higher IC50 of 5-fluorouracil (Fig. 6A). This result indicated the chemotherapy regimen based on 5-fluorouracil had a better effect in the low-risk score group. Compared with CRC samples with high PRS, samples with low PRS were more sensitive to anti-PD1 immunotherapy (Bonferroni corrected *p* < 0.01) (Fig. 6B). IMvigor 210 is the phase II trial of atezolizumab in patients with locally advanced or metastatic urothelial bladder cancer, and atezolizumab is an engineered anti-PDL1 antibody [23]. In IMvigor 210 cohort, we found that PRS of patients with “inflamed” immune phenotype was significantly lower than those with “desert” and “excluded” immune phenotypes (Fig. 6C). Further, patients with response to atezolizumab (n = 68) had significantly lower PRS than those who had no response (n = 230) (Fig. 6D). Moreover, this study predicted some potential compounds/drugs for CRC samples with different PRS via CTRP and PRISM. According to the correlation between compounds/drugs and the PRS of CRC samples, top 6 compounds/drugs with positive or negative correlation coefficients were screened out as potential drugs for the treatment of CRC with high PRS or low PRS, respectively. In CTRP, KU 0060648, BRD-K50799972, C6-ceramide, VU0155056, BRD-K13999467, BRD-K52037352 were top 6 potential drugs for CRC with high PRS. Drugs such as sildenafil and temozolomide could be used as potential drugs for CRC with low PRS (Fig. 6D). In PRISM, some drugs such as noretynodrel and isofloxythepin was predicted as potential drugs to treat CRC with high PRS, while temocapril, ethinyl-estradiol, etc. may be helpful in the treatment of CRC with low PRS (Fig. 6E).

### Exploration of pyroptosis-related risk model in the level of single-cell

**Fig. 7:**
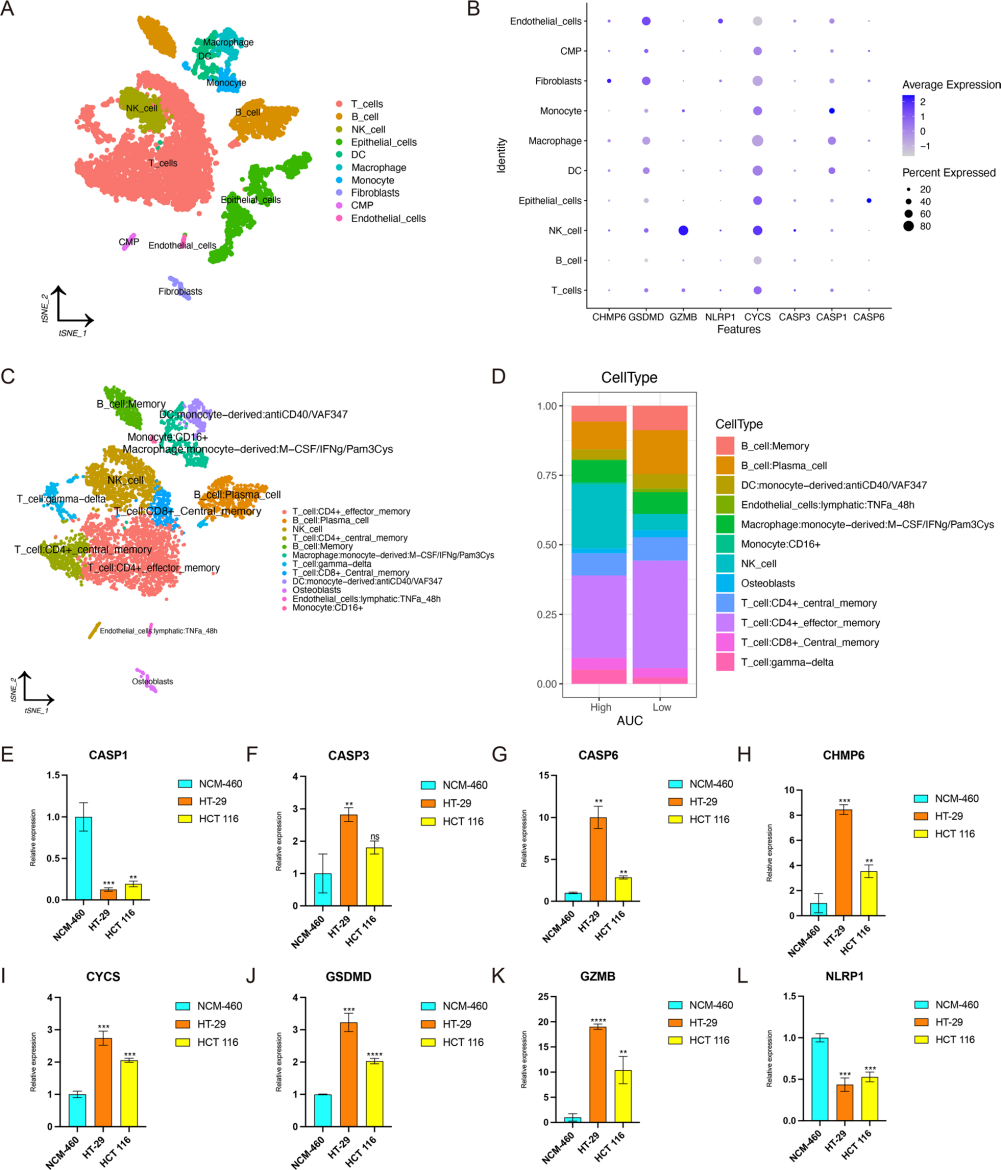
Exploration of pyroptosis-related genes by scRNA-seq data and RT-qPCR. **A** Cell annotation of clusters identified by t-SNE. **B** The expression of pyroptosis-related genes in cell subsets. **C** Cell annotation of immune cell subsets. **D** The proportion of different cell subsets in the low- and high-AUC groups. **E**–**L** Comparison of expression levels of genes between normal and CRC cell lines. **E** CASP1. **F** CASP3. **G** CASP6. **H** CHMP6. **I** CYCS. **J** GSDMD. **K** GZMB. **L** NLRP1. The top of error bar represents Mean + SD. The bottom of error bar represents Mean-SD. Lightblue represents NCM-460, coral represents HT-29, and yellow represents HCT 116. And ns means no significance; * means p < 0.05; ** means p < 0.01; *** means p < 0.001; **** means p < 0.0001

We collected scRNA-seq data of 5 CRC samples from GSE132257 data set (Additional file 1: Fig. S7A). After data filtering and standardization, 3000 genes with the largest variance were selected for subsequent cell classification (Additional file 1: Fig. S7B). The expression levels of these 3000 genes were dimensionally reduced to PC1-20 by PCA (Additional file 1: Fig. S7C). Then, t-distributed stochastic neighbor embedding (t-SNE) was performed on PC1-20 to classify all cells into 21 clusters (0–20; Additional file 1: Fig. S7D). The expression level of each pyroptosis-related gene in different clusters was displayed in Additional file 1: Fig. S8A. We then performed cell annotation for each cluster. T cells, B cells, natural killer (NK) cell, epithelial cells, dendritic cells (DC), macrophage, monocyte, fibroblasts, common myeloid progenitor (CMP), as well as endothelial cells were main cell types in these clusters (Fig. 7A). The expression and percentage of pyroptosis-related genes in different cell subsets were displayed in Fig. 7B. In addition, AUCell scoring algorithm was applied to assign all cells into low- and high-AUC groups. We found two peaks in the AUC values of all cells, while 4614 cells showed relatively higher AUC values when the AUC value threshold was set to 0.039 (Additional file 1: Fig. S8B). And Additional file 1: Fig. S8C showed AUC value of each cell. In order to further investigate the proportion of immune cell subsets in the low- and high-AUC groups, we provided more detailed annotations for immune cell subsets, which was displayed in Fig. 7C. And the proportion difference of immune cell subsets between the low- and high-AUC groups was shown in Fig. 7D. Compared to high-AUC group, the proportion of B cell, T cell and DC in the low-AUC gourp was higer, which might account for, at least part of the reason that samples with low PRS were more sensitive to anti-PD1 immunotherapy.

### Comparison of the expression levels of pyroptosis-related genes between normal and CRC cell lines

To further validate the clinical practicability of pyroptosis-related risk model, we performed RT-qPCR to quantify the expression levels of these 8 genes of the risk model in normal (NCM-460) and CRC cell lines (HT-29, HCT 116). Figure 7E–L showed the relative expression of each gene, which was represented by the mean with standard deviation (SD). The top of error bar represents Mean + SD. The bottom of error bar represents Mean-SD. Lightblue represents NCM-460, coral represents ﻿HT-29, and yellow represents ﻿HCT 116. And ns means no significance; * means p < 0.05; ** means p < 0.01; *** means p < 0.001; **** means p < 0.0001. Compared with NCM-460, CASP1, NLRP1 were down-regulated in both HT-29 and HCT 116. Reversely, CASP3, CASP6, CHMP6, CYCS, GSDMD, GZMB were up-regulated in both HT-29 and HCT 116.

### Consensus clustering

**Fig. 8:**
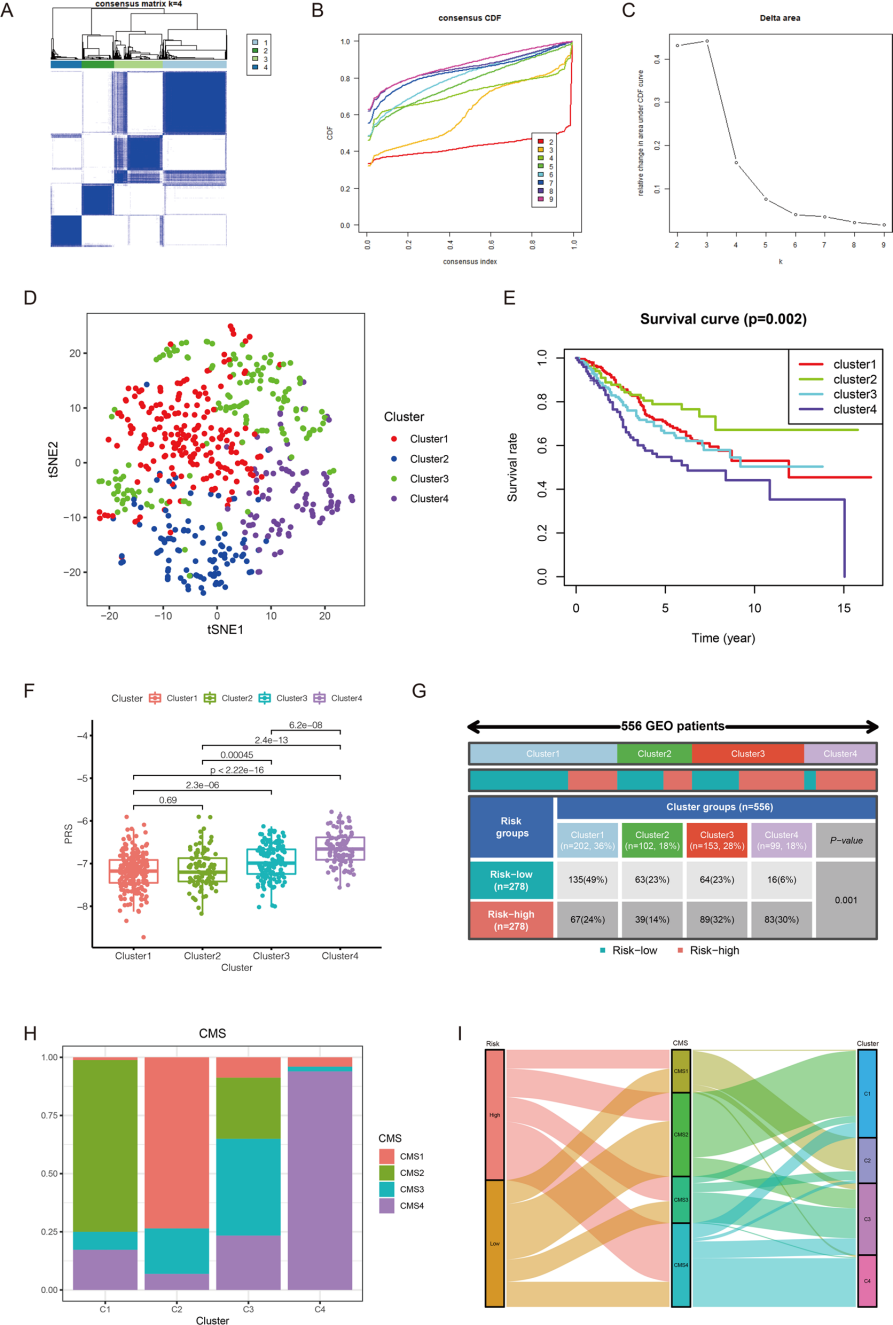
Consensus clustering and CMS subgroups. **A** CRC samples in the GEO cohort are clustered into 4 clusters. **B** Cumulative Distribution Function (CDF) of consensus clustering. **C** The relative change of the area under the CDF curve for different number of clusters. X-axis represents the number of clusters; Y-axis represents relative change in area under CDF curve. **D** The t-SNE of CRC samples in 4 clusters. **E** Survival analysis of CRC samples in 4 clusters. **F** The comparison of PRS in 4 clusters. **G** High- and low-risk score sample proportion among different clusters. **H** CMS subgroup proportion among different clusters. **I** The changes of PRS, CMS and clusters in individual samples displayed in alluvial diagram

In this study, 6719 DEGs with FDR < 0.01 were identified between high- and low-risk score groups. As shown in Additional file 1: Fig. S9A, top 20 up-regulated DEGs and down-regulated DEGs in CRC samples with high PRS were displayed in the form of heat map. Based on these DEGs, CRC samples in the GEO cohort were clustered into 4 clusters, including 202 samples in cluster1, 102 samples in cluster2, 153 samples in cluster3, 99 samples in cluster4 (Fig. 8A). Figure 8B showed the corresponding curves of Cumulative Distribution Function (CDF) when the number of clusters was 2–9. According to Fig. 8C, we could find that when k = 4 is the most significant inflection point of the CDF area change. The result of t-SNE showed that four clusters had prominent distinctions, indicating samples in different clusters may have different prognosis and other biological features (Fig. 8D). Survival analysis revealed that cluster2 had prominent survival advantage and cluster4 had worse prognosis compared with other clusters (Fig. 8E). Furthermore, the comparison of PRS between these clusters illustrate that cluster4 had significantly higher PRS than other clusters (Fig. 8F). As shown in Fig. 8G, 83 out of 99 samples (84%) in cluster4 were from the high-risk score group. In contrast, only a small part of the samples in cluster1 (67; 33%) and cluster2 (39; 38%) were from the high-risk score group. These results explained why samples in cluster4 had the worst prognosis. Besides, we explored the relation between these four clusters and CMS subgroups. As shown in Fig. 8H, we inferred that cluster1 corresponded to CMS2, cluster2 corresponded to CMS1, cluster3 corresponded to CMS3 and cluster4 corresponded to CMS4. The changes of PRS, CMS and clusters in individual samples were visualized by the alluvial diagram (Fig. 8I).

**Fig. 9:**
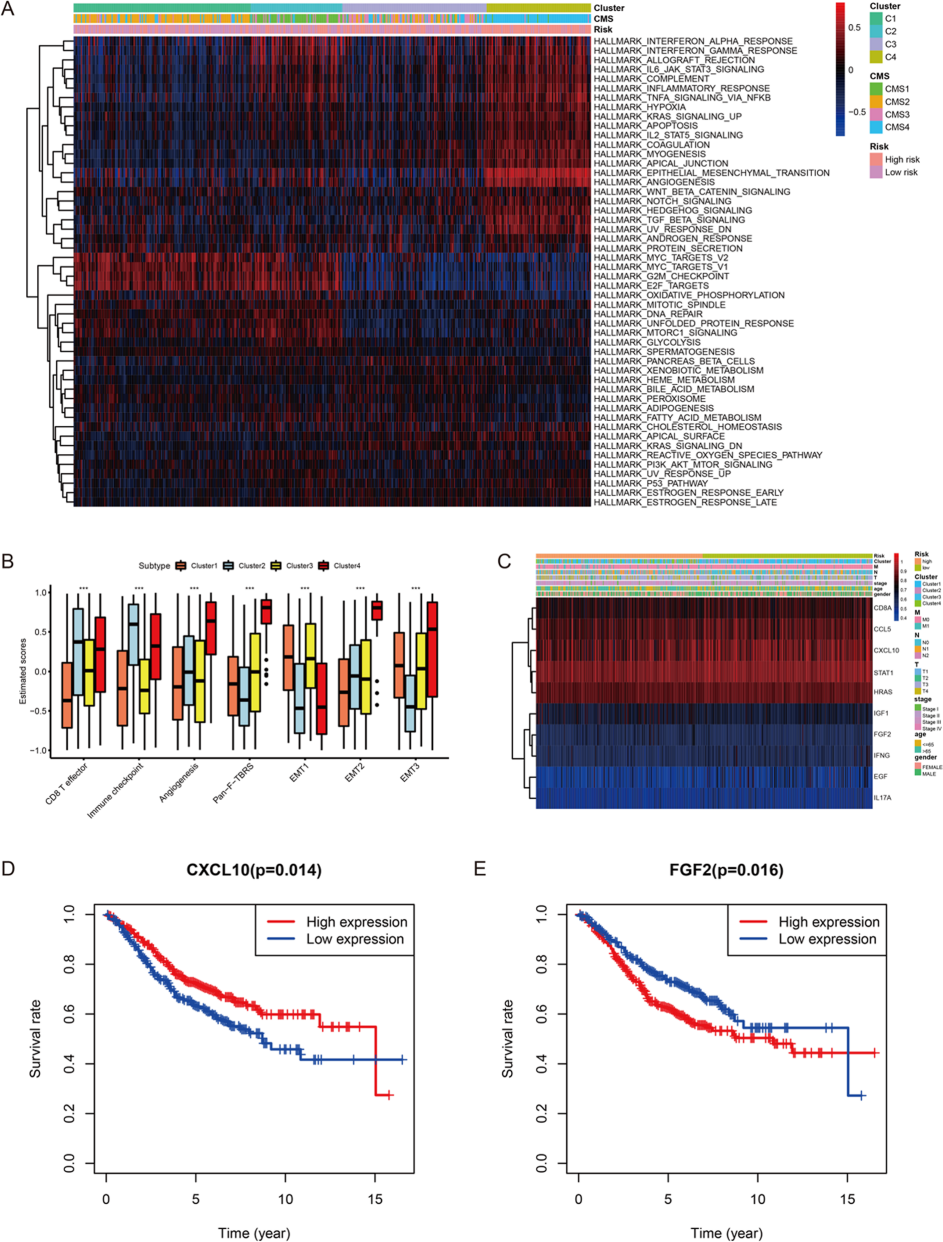
The exploration of biological characteristics between 4 clusters. **A** The results of GSVA among 4 clusters. **B** The comparison of immune-related and stroma-related activities in 4 clusters. **C** The expression levels of 10 hub genes in high- and low-risk score groups and clinical subgroups. **D** Survival analysis between low-expressed CXCL10 and high-expressed CXCL10 groups. **E** Survival analysis between low-expressed FGF2 and high-expressed FGF2 groups

In order to have a deeper understanding of the characteristics of samples in different clusters, GSVA was conducted to evaluate the enrichment of the biological functions and immune-related activities. The results of GSVA revealed that cluster2 and cluster4 were significantly related with immune-related activities. However, stromal activation such as EMT and angiogenesis were also enriched in cluster4. Biological metabolism such as xenobiotic metabolism, bile acid metabolism, as well as fatty acid metabolism were observed to be enriched in cluster3. And some canonical tumor pathways were activated in cluster1 (Fig. 9A). Subsequent analysis revealed that CD8 T effector and immune checkpoint were significantly activated in cluster2; stromal activities, including angiogenesis, Pan-F-TBRS and EMT1/2/3, were prominently enhanced in cluster4 (Fig. 9B). These results further confirmed the correspondence between clusters and CMS subgroups.

### Hub genes between high- and low-risk score groups

Total 338 overlapping genes were determined for subsequent PPI network construction from DEGs and immune genes (Additional file 1: Fig. S9B). PPI network of these overlapping genes was displayed in the Additional file 1: Fig. S9C. The color of gene represents the PPI score of gene (indicating the degree of interaction), the closer to red the higher the score, and the closer to purple the lower the score. Up-regulated genes in the high-risk score group were marked with red, down-regulated genes in the high-risk score group were marked with blue (Additional file 1: Fig. S9D). According to the degree, STAT1, FGF2, HRAS, IGF1, CXCL10, CD8A, EGF, IL17A, CCL5, as well as IFNG were considered as top 10 hub genes (Additional file 1: Fig. S9E). The interaction of these 10 hub genes were demonstrated in Additional file 1: Fig. S9F. The association between hub genes and immune cells was investigated, and Additional file 1: Fig. S9G showed the expression level of hub genes was closely related to immune cells infiltration. In addition, the expression levels of these hub genes in high- and low-risk score groups and clinical subgroups were displayed in the Fig. 9C. Upon further analysis, we found the CXCL10 and FGF2 expression level played important roles in CRC samples’ survival. Compared with the high CXCL10-expressed samples, the low CXCL10-expressed samples had poorer prognosis (Fig. 9D). On the contrary, CRC samples with low FGF2 expression had better prognosis than that with high FGF2 expression (Fig. 9E). We also found that the expression levels of CXCL10 and FGF2 were positively related with the infiltration of immune cells such as B cells, CD8+ T cells, CD4+ T cells, and dendritic cells in CRC (Additional file 1: Fig. S10A, B).

## Discussion

Pyroptosis is a kind of inflammatory PCD triggered by caspase-1/4/5/11 that is activated by some inflammasomes [24]. These inflammasomes are multi-protein complexes containing pattern-recognition receptors (PRR), which can recognize certain pathogen-associated molecular patterns (PAMPs) caused by invading pathogens and damaged-associated molecular patterns (DAMPs) from endogenous pathogens, respectively [25, 26]. There are two ways to trigger pyroptosis, one is the canonical inflammasome pathway via caspase-1 activation, the other is the non-canonical inflammasome pathway by the activation of caspase-4/5 and caspase-11 [27–29]. Since the activation of pyroptosis by inflammasome pathways is partly mediated by the caspase substrate Gasdermin-D (GSDMD), pyroptosis is also called as GSDM-mediated programmed necrotic cell death [5, 30, 31]. In recent, some studies have pointed out that exogenously activated pyroptosis could arouse robust anti-tumor activity in many types of malignant tumors such as digestive cancers, respiratory cancers and reproductive cancers [32–36]. For instance, the study of Wang et al. revealed that GSDMD, executioner of pyroptosis, played an important role in preventing gastric cancer (GC) cells from proliferation [37]. In colitis-associated colorectal cancer (CAC), many studies found that the expression level of components of inflammasome such as NLRP3 decreased in the CAC mouse model [38, 39]. Moreover, emerging studies have indicated that pyroptosis is related to the activation of anti-tumor immunity [40, 41]. Emerging experiments and clinical studies have found that immunotherapy does have advantages that traditional anti-tumor treatments cannot match, which can improve OS and PFS [42]. The mechanism of immunotherapy is mainly to strengthen the immune system by adjusting the immune microenvironment so that immune cells can attack and eliminate tumor cells on several key nodes [43]. Therefore, we inferred that exploring the association between pyroptosis and CRC contributed to guiding the treatment of CRC more effectively.

In this study, pyroptosis in CRC samples was quantified by the corresponding PRS calculated from pyroptosis-related risk model. Several genes in this model have been shown to play important roles in the induction of pyroptosis and immunity. For example, when granzyme B (coded by GZMB) cleaves and activates GSDME, NK and CD8+ T cells and chimeric antigen receptor (CAR) T cells can directly induce pyroptosis in GSDM-expressing tumors [44]. Beyond that, NLRP1 is the first member of the NLR family identified to form an inflammasome complex, playing a role in driving pyroptosis by catalyzing the proteolytic cleavage of pro-interleukin-1β (pro-IL-1β) and pro-IL-18 [45, 46]. In terms of prognosis, CRC samples with higher PRS had worse OS and PFS than that with lower PRS, indicating that PRS could act as an indicator for distinguishing survival of CRC. And the result of distribution of PRS in different clinical subgroups revealed that CRC samples at advanced stage had higher PRS compared with the early CRC samples, which was consistent with the above result, indicating PRS could reflect the development of CRC. Beyond that, the validation of expression levels of pyroptosis-related genes by RT-qPCR further demonstrated the feasibility of the risk model in clinical application. To sum up the above, PRS may be used as a supplementary tool to better assist clinical diagnosis and treatment of CRC. In addition to the prognosis, CRC samples with different PRS also had other distinct biological characteristics. Compared with high-risk score group, the expression of immune-related genes was higher in low-risk score group, such as PD-1, PD-L1 and CTLA4. Currently, immune checkpoint inhibitors (ICIs) targeting CTLA4 or PD-1/L1 have been shown to provide survival advantage in some treatment-refractory malignant tumors [47]. Moreover, the studies of Cogdill et al. have revealed the abundance of tumor-infiltrating lymphocytes (TILs) and other immune cells in the TME can affect the response to ICIs therapy [48]. Increased TILs in tumor can be a main hallmark of immunoinflammatory phenotype, exhibiting enhanced immune-mediated elimination of tumor cells [49]. In this study, we founded that activated CD8+ T cell, activated CD4+ T cell, as well as activated B cell were highly infiltrated in the CRC samples with low PRS, indicating PRS might be used to predict the response of CRC to immunotherapies. In CRC, two anti-PD-1 antibodies, nivolumab and pembrolizumab, proved to be effective in CRC patients with dMMR, and have received regulatory approval for the treatment of CRC that is dMMR [50]. Interestingly, there was also significant difference of PRS between MMR subgroups, CRC samples in dMMR subgroup had lower PRS compared with pMMR subgroup. Combined with the above results, we inferred that CRC samples with low PRS were more sensitive to immunotherapies than that with high PRS.

To further explore whether pyroptosis-related risk model could distinguish CRC samples that have different responses to drug treatment, we compared the response of samples in the high- and low-risk score groups to chemotherapy and immunotherapy, respectively. As expected, CRC samples in the low-risk score group were more sensitive to 5-fluorouracil and anti-PD-1 immunotherapy. This confirmed our hypothesis and illustrated pyroptosis-related risk model could be used as a tool to screen CRC samples suitable for chemotherapy and immunotherapy. However, due to the heterogeneity of CRC, not all CRC samples respond to existing ICIs, so it is particularly urgent to find potential compounds/drugs for personalized treatment. In this study, we predicted some compounds/drugs as potential targets for the treatment of CRC with different PRS. For example, C6-ceramide was predicted as a potential drug for CRC samples with high PRS. Based on cell experiments, C6-ceramide has been shown to individually inhibit the growth of both *KRAS* wild-type and *KRAS* mutant CRC cell lines (SW48 and SW480, respectively), especially in SW480 cell line. It is worth noting that oxaliplatin, cetuximab, and 5-FU combined with C6-ceramide increase the inhibition rate in both two CRC cell lines, and the inhibitory effect on *KRAS* mutant cells is particularly significant [51]. Therefore, C6-ceramide may revert the sensitivity of CRC samples with high PRS to drug treatment, even with *KRAS* mutation.

A drawback of bulk-sample expression analysis is that it may classify cell-type-specific genes as dysregulated in cancer only due to systematic differences in cell composition. In order to correct potential bias of clustering results generated from bulk sequencing data and verify our theory, single-cell analysis was performed to gain a ‘high resolution’ insight into how pyroptosis-related genes modify TME of CRC. This study revealed different cell subsets had distinct expression levels of pyroptosis-related genes. The proportion of T cell, B cell, and DC was higher in the low-AUC group, explaining why CRC samples with different PRS have different immune characteristics and responses to immunotherapy to some extent.

Based on DEGs between high- and low-risk score groups, CRC samples were classified into four clusters with significant prognostic differences. To enhance our understanding of tumor heterogeneity in CRC, we explored the activation of immune, stroma, metabolism, and tumor pathways among the four clusters. CRC samples in cluster2 was characterized by the activation of immunity, corresponding to CMS1. Although there was immune activation in cluster4, stromal activities such as TGF-β, EMT and angiogenesis were also activated, corresponding to CMS4. CRC samples in cluster1 was characterized by the activation of canonical tumor pathways, corresponding to CMS2. And CRC samples in cluster3 was characterized by the activation of metabolism, corresponding to CMS3. Guinney et al. established four CMS subgroups with distinguishing features: CMS1 is regarded as microsatellite instability immune; CMS2 is regarded as canonical, marked with WNT and MYC signaling activation; CMS3 is regarded as metabolic with evident metabolic dysregulation; and CMS4 is regarded as mesenchymal, with TGF-β activation, stromal invasion and angiogenesis. Finally, 2 prognostic hub genes, CXCL10 and FGF2, were identified. The higher expression of CXCL10 indicated the better OS. On the contrary, the prognosis of CRC samples with higher expression of FGF2 was worse. We also found that there were positive relations between infiltration level of CD8+ T cell and expression level of these 2 prognostic hub genes. CXCL10, encoding chemokines of the CXC subfamily and ligand for the receptor CXCR3, plays a role in some biological activities, which are relevant to various human diseases, such as dysfunction of immune and tumor development [52, 53]. Recent study indicated that under-expression of CXCL10 was related with tumor metastasis and poorer OS [54]. FGF2, a main component necessary for self-renewal of pluripotent stem cells, acts as a risk factor in CRC prognosis [55, 56]. These conclusions are consistent with our results.

There are still some limitations in this study. First, the analytical data for this study were derived from public databases, and all samples selected in our study were obtained retrospectively. Hence, inherent samples selection bias may affect the results. Some important clinical variables, such as neoadjuvant chemotherapy, chemoradiotherapy, and genetic mutation information, were not complete in all samples, which may have an impact on the prognosis, immune response, and pyroptosis of CRC. This study also only verified the expression differences of model genes in normal and CRC cell lines. The detailed mechanism by which pyroptosis alters the TME in CRC and thus affects prognosis and drug response needs to be explored and verified by prospective studies and other experimental studies in *vitro* and in *vivo*.

## Conclusion

The PRS, calculated by pyroptosis-related risk model, was correlated with pathological stages, AJCC-TNM stages and prognosis of CRC samples. Moreover, CRC samples with different PRS differ in immune and stromal characteristics, which in turn show different sensitivity to chemotherapy and immunotherapy. These findings indicate the crucial role of PRS in CRC, providing a novel and efficient perspective for tumor prognosis and treatment prediction.

## Supplementary Information

**Additional file 1: Figure S1.** The results of principal component analysis (PCA). (A) PCA before reducing batch effect of GEO and TCGA RNA-seq by sva. (B) PCA after reducing batch effect of GEO and TCGA RNA-seq by sva. (C) PCA before LASSO Cox regression analysis in the GEO cohort. (D) PCA after LASSO Cox regression analysis in the GEO cohort. (E) PCA before LASSO Cox regression analysis in the TCGA cohort. (F) PCA after LASSO Cox regression analysis in the TCGA cohort. **Figure S2.** The differences of prognosis between high- and low-risk score groups in the validation group. (A) The comparison of OS between high- and low-risk score groups. (B) The comparison of PFS between high- and low-risk score groups. (C) The ROC curves for OS prediction by PRS at 1-, 3- and 5-year. (D) The ROC curves for PFS prediction by PRS at 1-, 3- and 5-year. **Figure S3.** The association between PRS and clinical features in the TCGA cohort. (A) The comparison of PRS in CRC samples’ age. (B) The comparison of PRS in CRC samples’ gender. (C) The comparison of PRS in CRC samples’ pathological stages. (D) The comparison of PRS in CRC samples’ AJCC-T stages. (E) The comparison of PRS in CRC samples’ AJCC-N stages. (F) The comparison of PRS in CRC samples’ AJCC-M stages. **Figure S4.** The association between PRS and clinical features in the GEO cohort. (A) The comparison of PRS between CIMP (-) and CIMP (+) subgroups. (B) The comparison of PRS between *BRAF* wild-type and *BRAF* mutant CRC samples. (C) The comparison of PRS between *TP53* wild-type and *TP53* mutant CRC samples. **Figure S5.** Prediction of CMS subgroups of CRC samples. (A) CMS prediction based on the RNA expression data in the GEO cohort. (B) CMS subgroups have significant differences in tumor biological characteristics. **Figure S6.** PRS as an independent prognostic indicator in the TCGA cohort. (A) Univariate Cox regression analysis to identify risk factors in CRC survival. (B) Multivariate Cox regression analysis to identify independent prognostic indicators in CRC. **Figure S7.** Preprocessing of scRNA-seq data in GSE132257. (A) Filtering and standardization of scRNA-seq data from GSE132257. (B) 3000 genes with the largest variance. (C) PCA analysis dimensionally reduces standardized scRNA-seq data to PC1-20. (D) Identification of cell clusters by t-SNE. **Figure S8.** Exploration of pyroptosis-related risk model in the level of single-cell. (A) The expression level of each pyroptosis-related risk model gene in different clusters. (B) AUC scoring of pyroptosis-related genes. The threshold was determined as 0.039 and the 4614 cells exceeded the threshold value. (C) The t-SNE plots based on the AUC score of cells. Cell subsets with high AUC score are highlighted. **Figure S9.** Protein–protein interaction (PPI) network. (A) Top 20 up-regulated DEGs and down-regulated DEGs between high- and low-risk score groups. (B) Overlapping genes between DEGs and immune genes. (C) PPI network of DEGs between high- and low-risk score groups by STRING. (D) PPI network processed by Cytoscape software. Red represents high-regulated gene in the high-risk score group. Blue represents down-regulated gene in the high-risk score group. (E) Top 10 hub genes identified by cytoHubba. The redder the color, the higher the degree of interaction between the gene and other genes. (F) Interaction between 10 hub genes. (G) The correlation between hub genes and immune cells. *p < 0.05; **p < 0.01. **Figure S10.** The correlation between gene expression and the infiltration of immune cells by TIMER. (A) The correlation between expression of *CXCL10* and the infiltration of immune cells. (B) The correlation between expression of *FGF2* and the infiltration of immune cells.

**Additional file 2: Table S1.** Clinical baseline of CRC samples.

## Data Availability

The public datasets were obtained from TCGA (https://www.cancer.gov/tcga) and GEO (https://www.ncbi.nlm.nih.gov/geo/).

## Abbreviations

CRC: Colorectal cancer
LASSO: Least Absolute Shrinkage and Selection Operator
PRS: Pyroptosis-related risk scores
TCGA: The Cancer Genome Atlas Program
GEO: Gene Expression Omnibus
OS: Overall survival
PFS: Progression-free survival
TME: Tumor microenvironment
RT-qPCR: Quantitative real-time polymerase chain reaction
ssGSEA: Single-sample gene-set enrichment analysis
TIDE: Tumor immune dysfunction and exclusion
PCD: Programmed cell death
IL: Interleukin
GI: Gastrointestinal
RNA-seq: RNA sequencing
scRNA-seq: Single-cell RNA sequencing
MSigDB: Molecular Signatures Database
PCA: Principal component analysis
ROC: Receiver operating characteristic
AUC: Area under the curve
EMT: Epithelial mesenchymal transition
CIMP: CpG island methylator phenotype
CIN: Chromosomal instability
MMR: DNA mismatch repair
CMS: Consensus Molecular Subtype
IC50: The half maximal inhibitory concentration
CCLs: Cancer cell lines
CCLE: Cancer Cell Line Encyclopedia
CTRP: Cancer Therapeutics Response Portal
PPI: Protein–protein interaction
GSVA: Gene set variation analysis
t-SNE: T-distributed stochastic neighbor embedding
GO: Gene ontology
KEGG: Kyoto Encyclopedia of Genes and Genomes
BP: Biological processes
CC: Cell component
MF: Molecular function
PRR: Pattern-recognition receptors
PAMPs: Pathogen-associated molecular patterns
DAMPs: Damaged-associated molecular patterns
GC: Gastric cancer
CAC: Colitis-associated colorectal cancer
ICIs: Immune checkpoint inhibitors
TILs: Tumor-infiltrating lymphocytes
