# Supplementary material for: Exploring the role of pyroptosis in shaping the tumor microenvironment of colorectal cancer by bulk and single-cell RNA sequencing
Source: Cancer Cell Int. 2023 May 18;23:95. doi: 10.1186/s12935-023-02897-8 (PMC10193704; doi:10.1186/s12935-023-02897-8)
Supplement: Supplementary file 1 — Additional file 1: Figure S1. The results of principal component analysis (PCA). (A) PCA before reducing batch effect of GEO and TCGA RNA-seq by sva. (B) PCA after reducing batch effect of GEO and TCGA RNA-seq by sva. (C) PCA before LASSO Cox regression analysis in the GEO cohort. (D) PCA after LASSO Cox regression analysis in the GEO cohort. (E) PCA before LASSO Cox regression analysis in the TCGA cohort. (F) PCA after LASSO Cox regression analysis in the TCGA cohort. Figure S2. The differences of prognosis between high- and low-risk score groups in the validation group. (A) The comparison of OS between high- and low-risk score groups. (B) The comparison of PFS between high- and low-risk score groups. (C) The ROC curves for OS prediction by PRS at 1-, 3- and 5-year. (D) The ROC curves for PFS prediction by PRS at 1-, 3- and 5-year. Figure S3. The association between PRS and clinical features in the TCGA cohort. (A) The comparison of PRS in CRC samples’ age. (B) The comparison of PRS in CRC samples’ gender. (C) The comparison of PRS in CRC samples’ pathological stages. (D) The comparison of PRS in CRC samples’ AJCC-T stages. (E) The comparison of PRS in CRC samples’ AJCC-N stages. (F) The comparison of PRS in CRC samples’ AJCC-M stages. Figure S4. The association between PRS and clinical features in the GEO cohort. (A) The comparison of PRS between CIMP (-) and CIMP (+) subgroups. (B) The comparison of PRS between BRAF wild-type and BRAF mutant CRC samples. (C) The comparison of PRS between TP53 wild-type and TP53 mutant CRC samples. Figure S5. Prediction of CMS subgroups of CRC samples. (A) CMS prediction based on the RNA expression data in the GEO cohort. (B) CMS subgroups have significant differences in tumor biological characteristics. Figure S6. PRS as an independent prognostic indicator in the TCGA cohort. (A) Univariate Cox regression analysis to identify risk factors in CRC survival. (B) Multivariate Cox regression analysis to identify independent prognostic [file 12935_2023_2897_MOESM1_ESM.pdf]

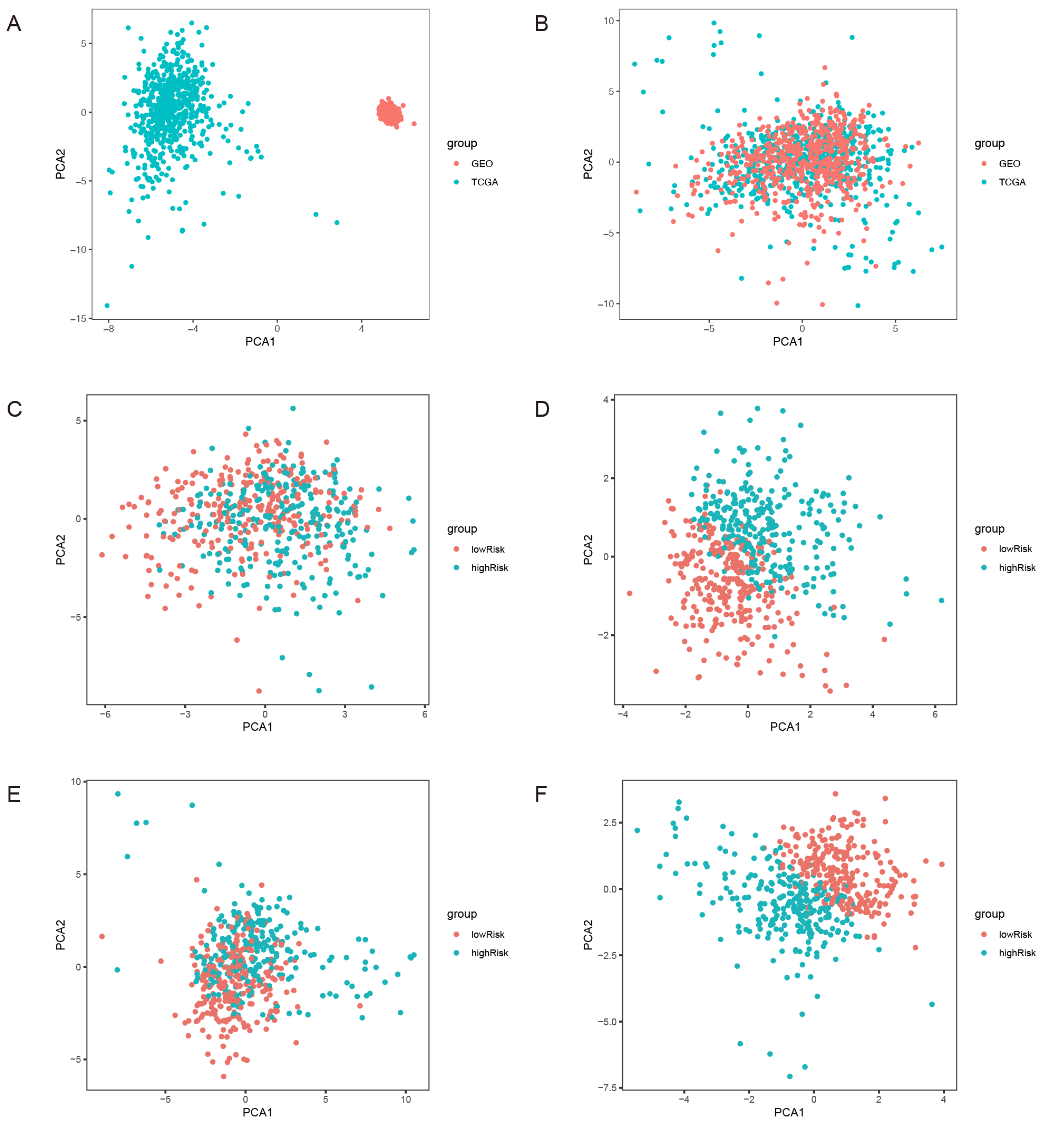

Supplementary figure 1. The results of principal component analysis (PCA). (A) PCA before reducing batch effect of GEO and TCGA RNA-seq by sva. (B) PCA after reducing batch effect of GEO and TCGA RNA-seq by sva. (C) PCA before LASSO Cox regression analysis in the GEO cohort. (D) PCA after LASSO Cox regression analysis in the GEO cohort. (E) PCA before LASSO Cox regression analysis in the TCGA cohort. (F) PCA after LASSO Cox regression analysis in the TCGA cohort.

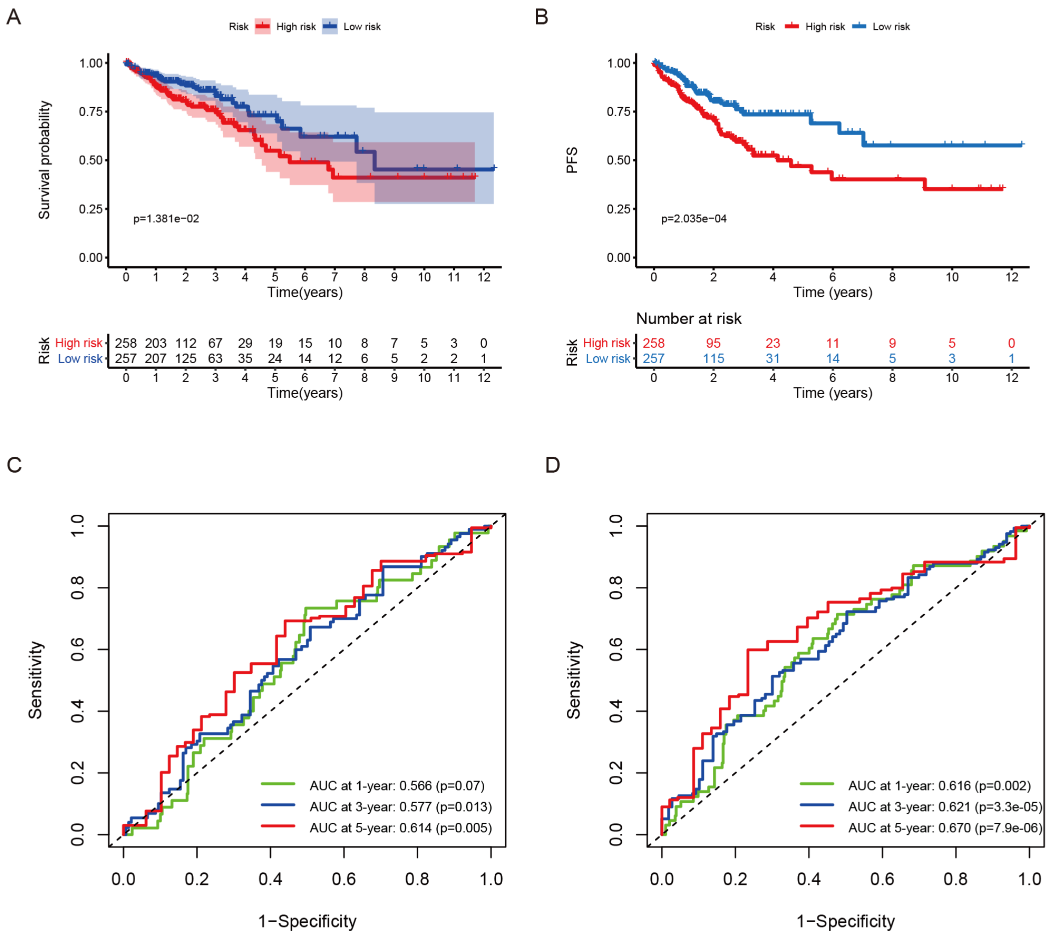

Supplementary figure 2. The differences of prognosis between high- and low-risk score groups in the validation group. (A) The comparison of OS between high- and low-risk score groups. (B) The comparison of PFS between high- and low-risk score groups. (C) The ROC curves for OS prediction by PRS at 1-, 3- and 5-year. (D) The ROC curves for PFS prediction by PRS at 1-, 3- and 5-year.

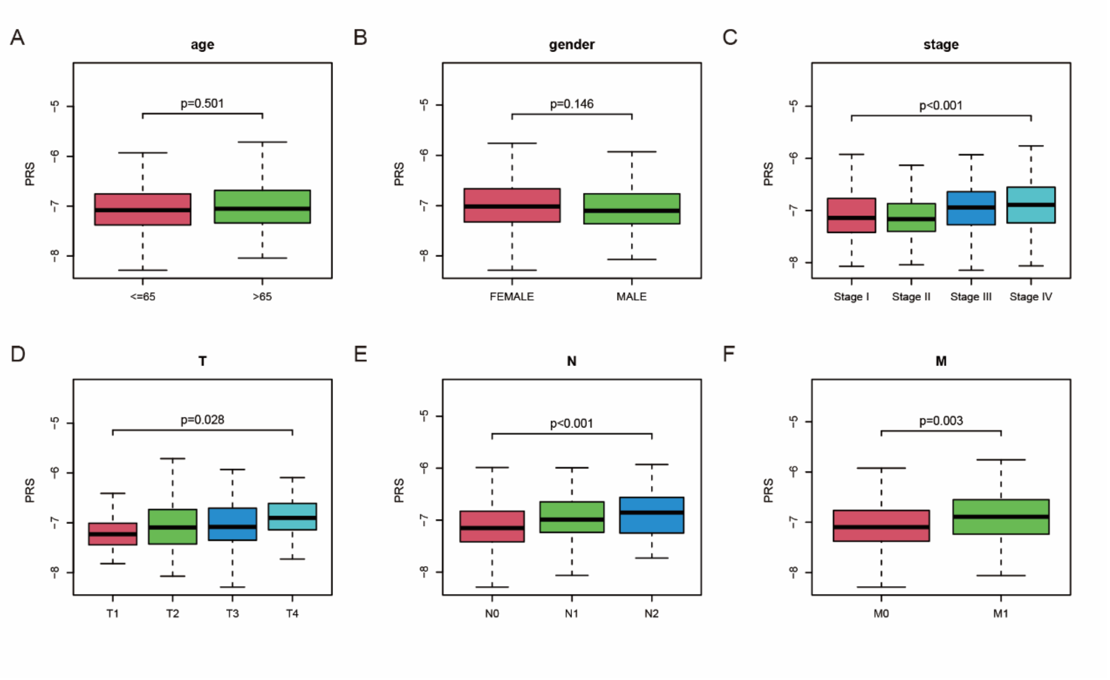

Supplementary figure 3. The association between PRS and clinical features in the TCGA cohort. (A) The comparison of PRS in CRC samples' age. (B) The comparison of PRS in CRC samples' gender. (C) The comparison of PRS in CRC samples' pathological stages. (D) The comparison of PRS in CRC samples' AJCC-T stages. (E) The comparison of PRS in CRC samples' AJCC-N stages. (F) The comparison of PRS in CRC samples' AJCC-M stages.

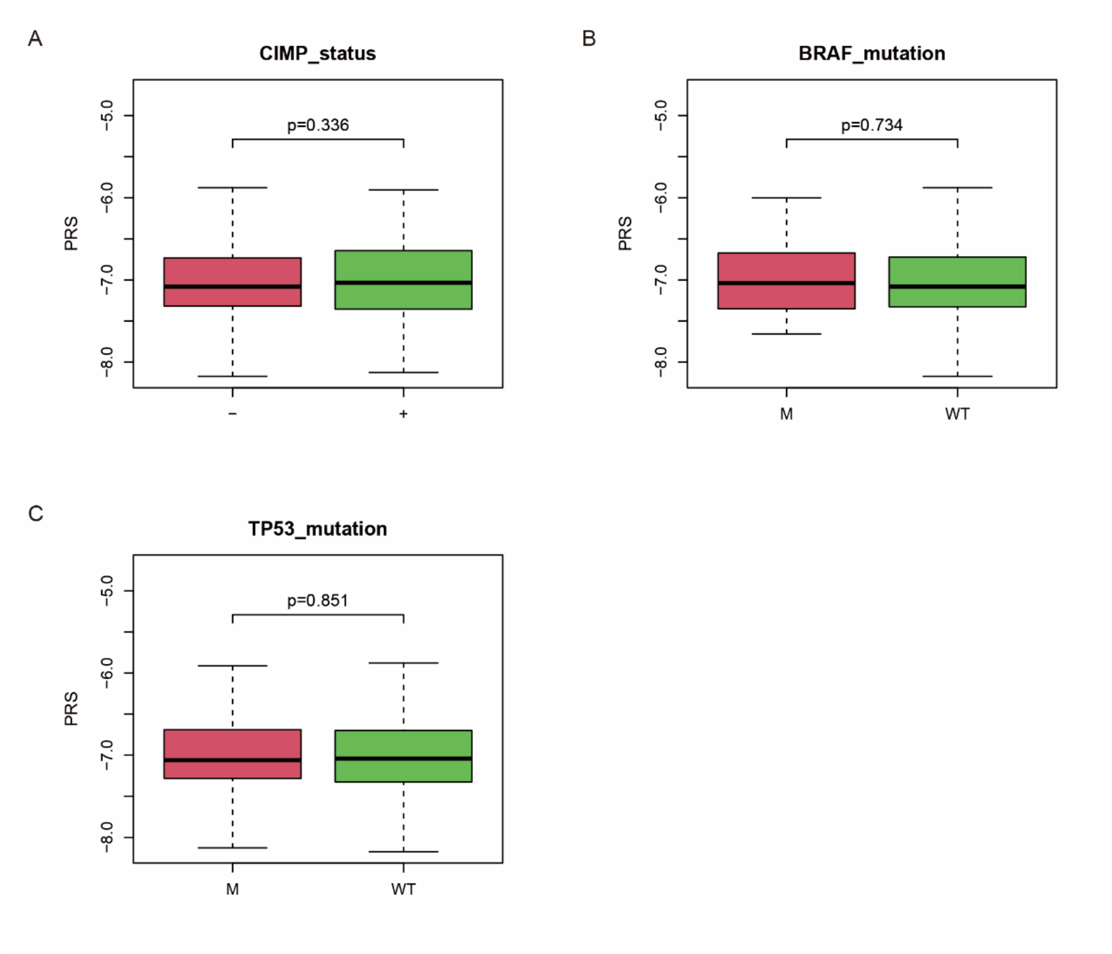

Supplementary figure 4. The association between PRS and clinical features in the GEO cohort. (A) The comparison of PRS between CIMP (-) and CIMP (+) subgroups. (B) The comparison of PRS between *BRAF* wild-type and *BRAF* mutant CRC samples. (C) The comparison of PRS between *TP53* wild-type and *TP53* mutant CRC samples.

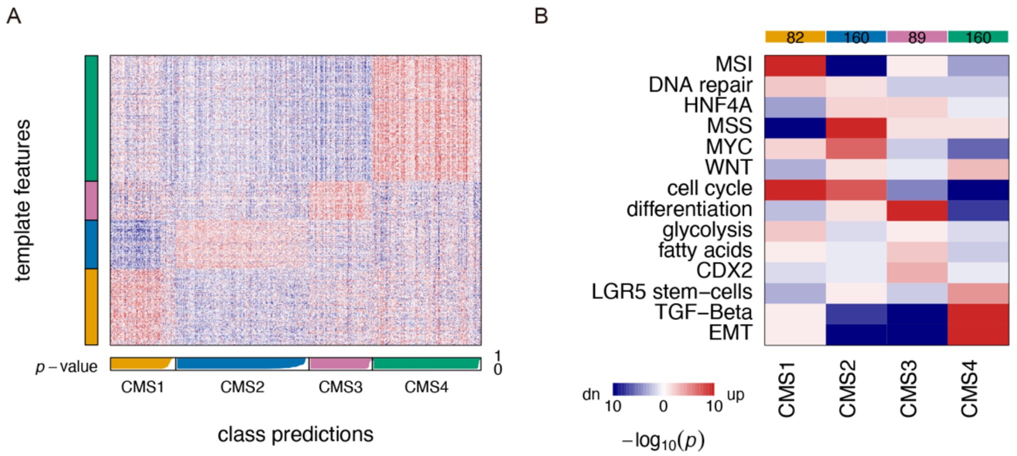

Supplementary figure 5. Prediction of CMS subgroups of CRC samples. (A) CMS prediction based on the RNA expression data in the GEO cohort. (B) CMS subgroups have significant differences in tumor biological characteristics.

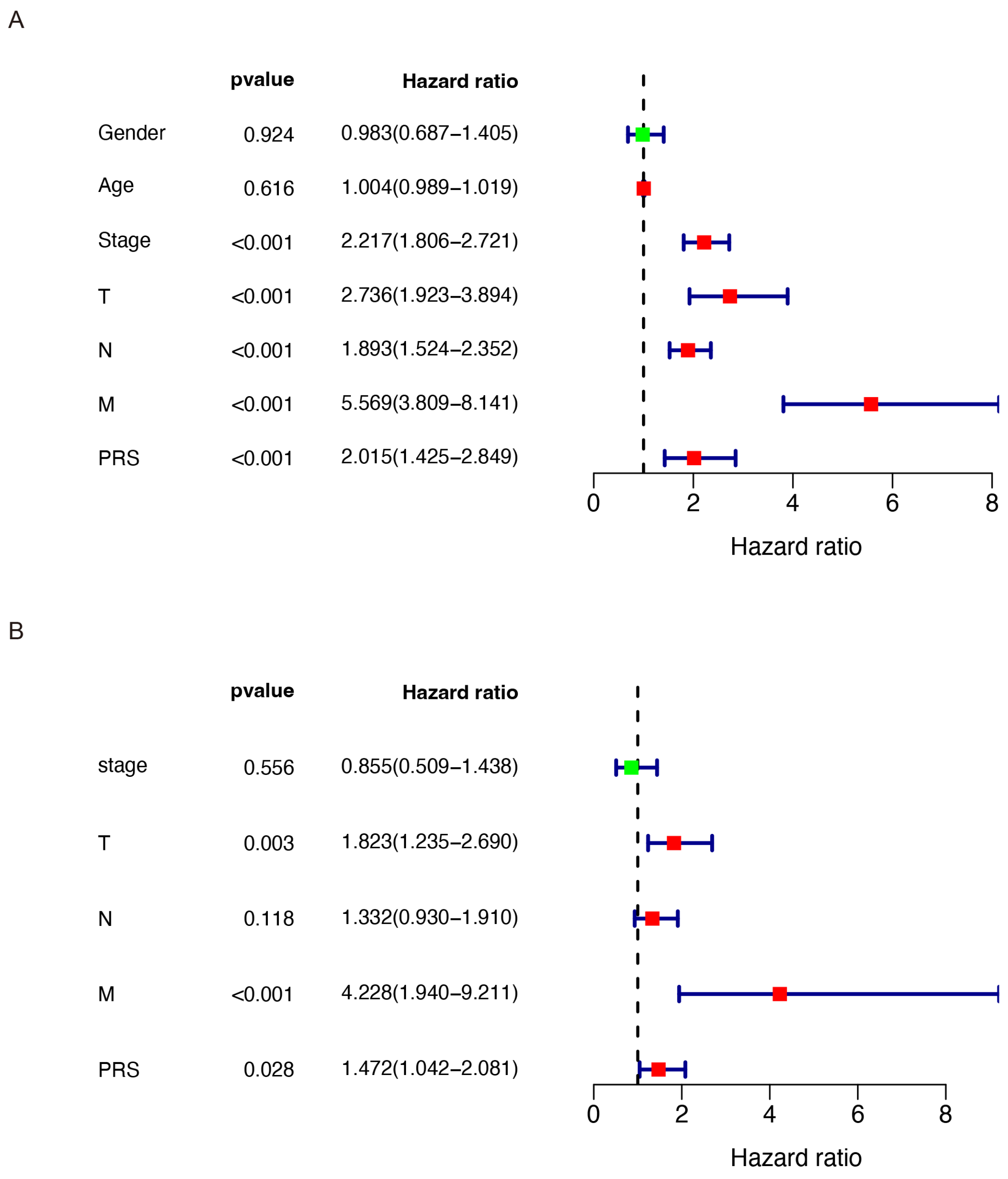

Supplementary figure 6. PRS as an independent prognostic indicator in the TCGA cohort. (A) Univariate Cox regression analysis to identify risk factors in CRC survival. (B) Multivariate Cox regression analysis to identify independent prognostic indicators in CRC.

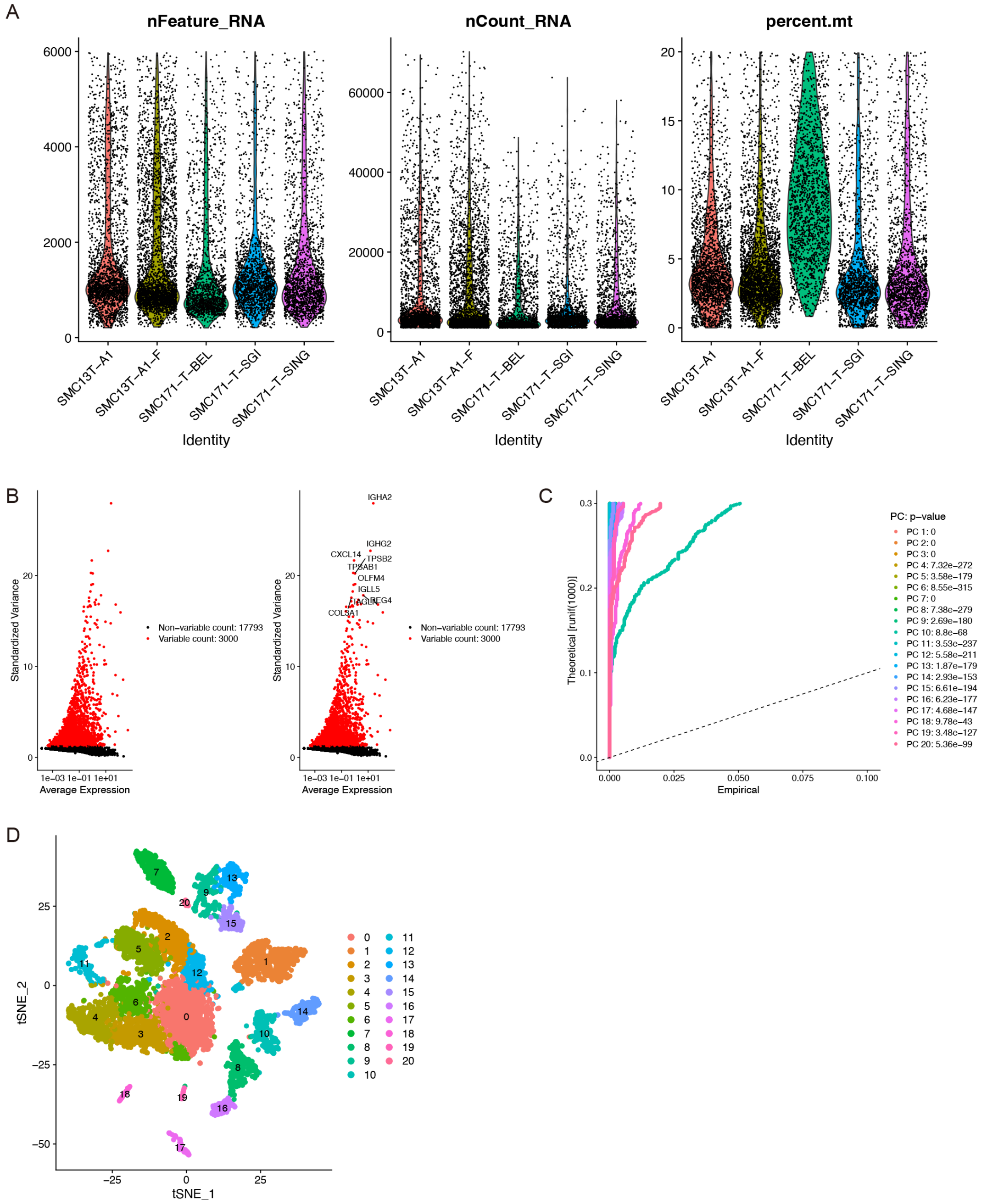

Supplementary figure 7. Preprocessing of scRNA-seq data in GSE132257. (A) Filtering and standardization of scRNA-seq data from GSE132257. (B) 3000 genes with the largest variance. (C) PCA analysis dimensionally reduces standardized scRNA-seq data to PC1-20. (D) Identification of cell clusters by t-SNE.

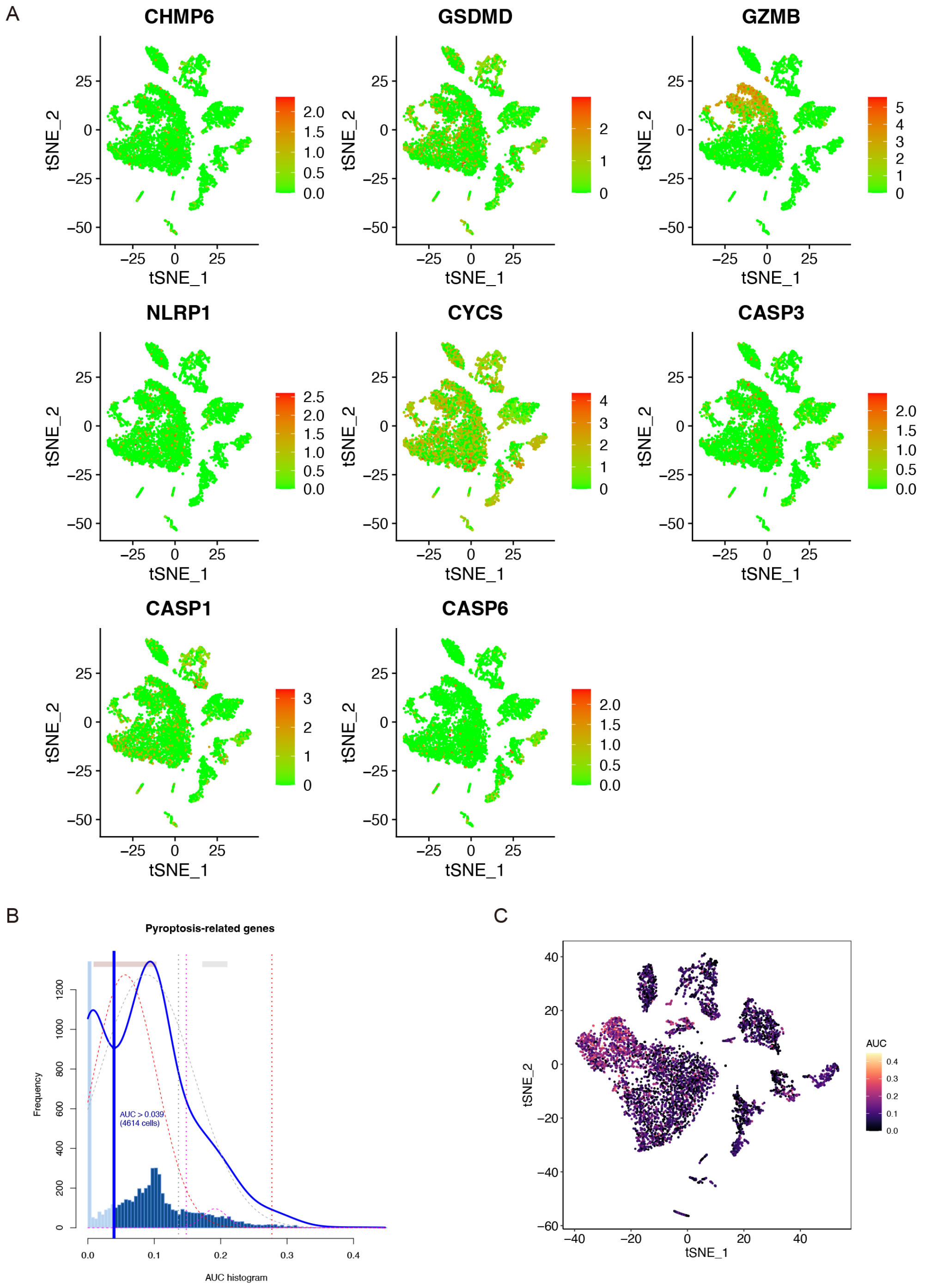

Supplementary figure 8. Exploration of pyroptosis-related risk model in the level of single-cell. (A) The expression level of each pyroptosis-related risk model gene in different clusters. (B) AUC scoring of pyroptosis-related genes. The threshold was determined as 0.039 and the 4614 cells exceeded the threshold value. (C) The t-SNE plots based on the AUC score of cells. Cell subsets with high AUC score are highlighted.

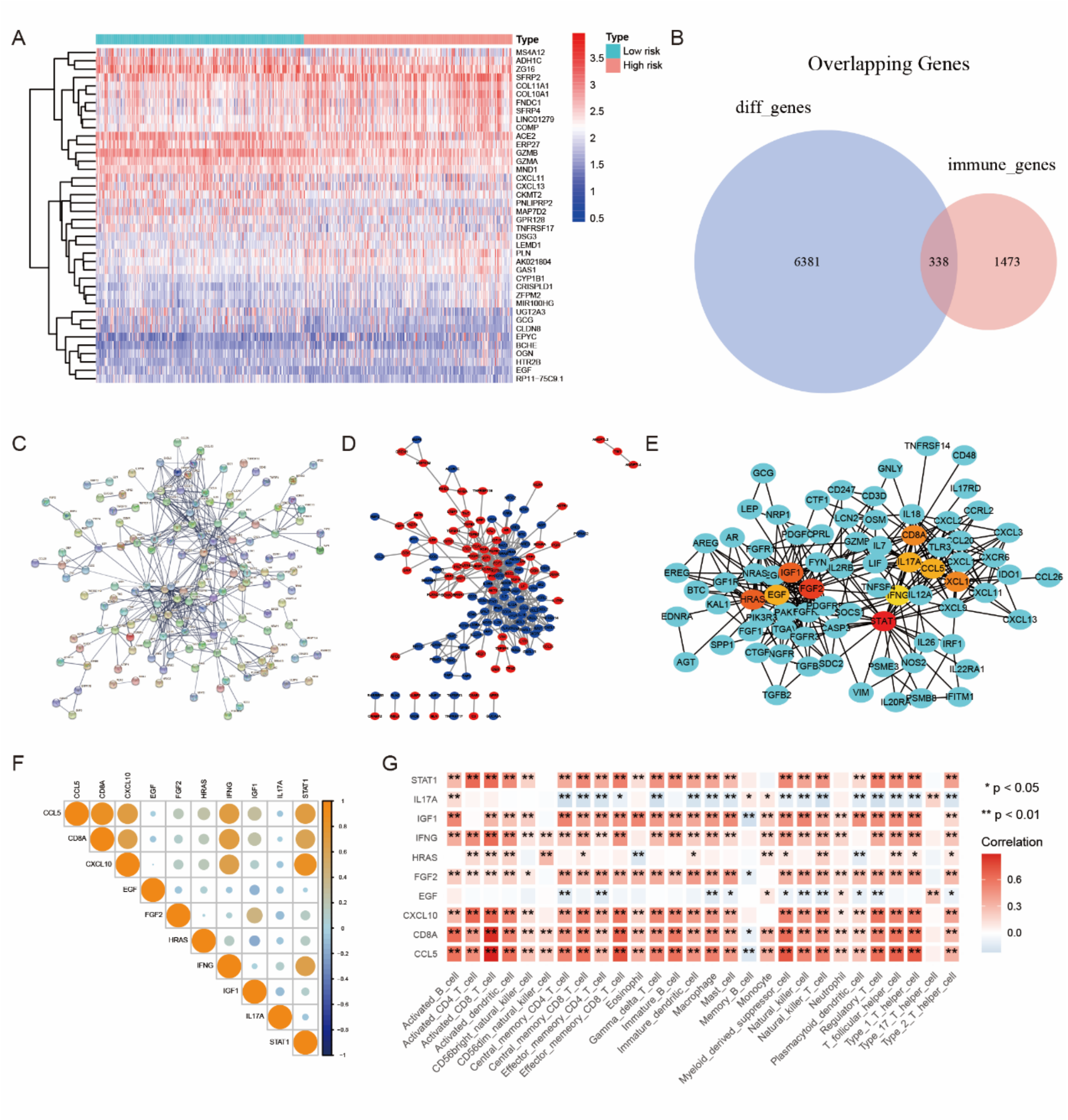

Supplementary figure 9. Protein-protein interaction (PPI) network. (A) Top 20 up-regulated DEGs and down-regulated DEGs between high- and low-risk score groups. (B) Overlapping genes between DEGs and immune genes. (C) PPI network of DEGs between high- and low-risk score groups by STRING. (D) PPI network processed by Cytoscape software. Red represents high-regulated gene in the high-risk score group. Blue represents down-regulated gene in the high-risk score group. (E) Top 10 hub genes identified by cytoHubba. The redder the color, the higher the degree of interaction between the gene and other genes. (F) Interaction between 10 hub genes. (G) The

correlation between hub genes and immune cells. * $p < 0.05$; ** $p < 0.01$.

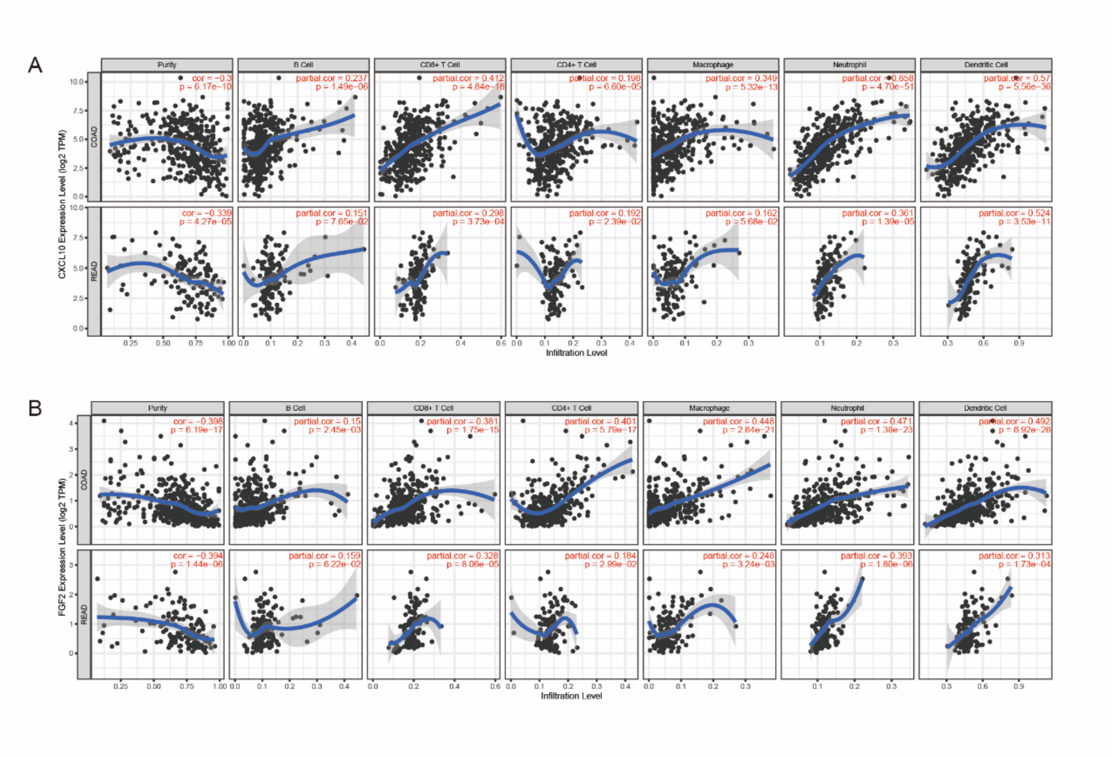

Supplementary figure 10. The correlation between gene expression and the infiltration of immune cells by TIMER. (A) The correlation between expression of *CXCL10* and the infiltration of immune cells. (B) The correlation between expression of *FGF2* and the infiltration of immune cells.
