# Supplementary material for: Exploring the role of pyroptosis in shaping the tumor microenvironment of colorectal cancer by bulk and single-cell RNA sequencing
Source: Cancer Cell Int. 2023 May 18;23:95. doi: 10.1186/s12935-023-02897-8 (PMC10193704; doi:10.1186/s12935-023-02897-8)
Supplement: Supplementary file 2 — Additional file 2: Table S1. Clinical baseline of CRC samples. [file 12935_2023_2897_MOESM2_ESM.pdf]

Supplementary table 1. Clinical baseline of CRC samples.

**Clinical information**	**GEO (training group)**	**TCGA (validation group)**
	n=556	n=515
**Age (years)**		
⩽ 65	222	228
> 65	333	287
Unknow	1	0
**Gender**		
Male	307	280
Female	249	235
**Pathological stage**		
I	32	91
II	258	193
III	203	142
IV	59	74
Unknow	4	15
**T**		
T1	11	15
T2	44	91
T3	360	351
T4	117	57
Unknow	24	1
**N**		
N0	295	301
N1	131	125
N2	104	88
Unknow	26	1
**M**		
M0	474	385
M1	60	73
Unknow	22	57
